# Autophagy induced by SAHA affects mutant P53 degradation and cancer cell survival

**DOI:** 10.1042/BSR20181345

**Published:** 2019-02-19

**Authors:** Giorgia Foggetti, Laura Ottaggio, Debora Russo, Carlotta Mazzitelli, Paola Monti, Paolo Degan, Mariangela Miele, Gilberto Fronza, Paola Menichini

**Affiliations:** Mutagenesis and Cancer Prevention Unit, IRCCS Ospedale Policlinico San Martino, Genoa 16132, Italy

**Keywords:** autophagy, apoptosis, mutant p53, SAHA

## Abstract

Missense mutations in the *TP53* gene produce mutant p53 (mutp53) proteins which may acquire oncogenic properties favoring chemoresistance, cell migration, and metastasis. The exploitation of cellular pathways that promote mutp53 degradation may reduce cell proliferation and invasion as well as increase the sensitivity to anticancer drugs, with a strong impact on current cancer therapies. In the last years, several molecules have been characterized for their ability to induce the degradation of mutp53 through the activation of autophagy. Here, we investigated the correlation between autophagy and mutp53 degradation induced by suberoylanilide hydroxamic acid (SAHA), an FDA-approved histone deacetylase inhibitor. In the human cancer lines MDA-MB-231 (mutp53-R280K) and DLD1 (mutp53-S241F), SAHA induced a significant mutp53 degradation. However, such degradation correlated with autophagy induction only in MDA-MB-231 cells, being counteracted by autophagy inhibition, which also increased SAHA-induced cell death. Conversely, in DLD1 cells SAHA triggered a low level of autophagy despite promoting a strong decrease in mutp53 level, and autophagy inhibition did not change either mutp53 levels or sensitivity to this drug. We conclude that autophagy can be a relevant pathway for mutp53 degradation induced by SAHA, but its contribution to mutp53 destabilization and the consequences on cell death are likely context-dependent.

## Introduction

The p53 tumor suppressor protein has a central role in cell homeostasis due to its involvement in many pathways that regulate cell cycle arrest and proliferation, cell response to stress, DNA repair, cell death and autophagy [1]. *TP53* is the most frequently mutated gene in human cancers and the presence of mutant p53 proteins (mutp53s) in tumors often correlates with a bad prognosis [2]. Mutp53 functions, not exhibited by the wild-type (wt) protein, promote malignancy and resistance to chemotherapy. These features, called gain of functions, were first demonstrated after the introduction of mutp53 in *TP53* null cancer cells [3]. Results obtained in mutp53 knockin mouse models showed that the stabilization of mutp53 is required for its oncogenic activity since, in these mice, mutp53 protein accumulated in tumors but its levels were found unstable in normal tissues [4–7]. Other studies have thoroughly demonstrated that the elimination of mutp53 decreases the proliferation of tumor cells, inhibits invasion and metastasis, and sensitizes tumor cells to genotoxic agents that are used in chemotherapy [8,9]. Thus, inducing mutp53 degradation would represent a useful therapeutic approach.

Recently, a class of molecules able to trigger mutp53 degradation through the induction of autophagy has been described. Amongst these, Zn(II)-compound and capsaicin have been shown to deplete the expression of mutp53 through autophagy stimulation [10–12]. We previously showed that PRIMA-1 (P53 Re-activation and Induction of Massive Apoptosis) triggers the degradation of mutp53 via ubiquitination [13] and that this activity correlates to autophagy induction [14]. We then demonstrated that Gambogic Acid (GA), a potent apoptotic molecule [15] that stimulates the degradation of mutp53 and increases the sensitivity of cancer cells to chemotherapeutic agents [16], induces mutp53 degradation through autophagy [17]. Other molecules able to trigger mutp53 degradation and sensitize cancer cells to cell death include: (i) Histone DeACetylases inhibitors (HDACi), for example suberoylanilide hydroxamic acid (SAHA) and (ii) heat shock protein 90 (HSP90) inhibitors such as 17-allylamino-17-demethoxygeldanamycin (17-AAG) [18] or ganetespid [19]. However, a different mechanism for mutp53 degradation has been demonstrated for these drugs [18,19].

SAHA, the first FDA-approved HDACi for the treatment of cutaneous T-cell lymphoma since 2006, is able to destabilize mutp53 through the inhibition of the HDAC6–HSP90 chaperone axis [18]. SAHA induces hyperacetylation of HDAC6 that, in turn, leads to hyperacetylation and consequent inhibition of HSP90. This post-translational modification leads to the dissociation of the HSP90–HDAC6–mutp53 complex, enabling the mutp53 degradation by the murine double minute 2 (MDM2)/C-terminus of Hsp70-interacting protein (CHIP) complex [18]. Besides this HDACi activity, it has been shown that SAHA has multiple cellular effects. For example, in cancer cells, SAHA can activate apoptosis, the accumulation of reactive oxygen species (ROS) and the activation of tumor necrosis factor α (TNFα) family members [20–22]. Furthermore, SAHA can induce autophagy [23–25].

Autophagy is a catabolic process in which damaged cellular proteins and cytoplasmic organelles are enclosed in double-membrane autophagic vesicles, called autophagosomes, that are targetted to lysosomes [26]. The fusion of autophagosomes with lysosomes results in the formation of autophagolysosomes, where the sequestered content is degraded and recycled for protein and ATP synthesis [26]. Autophagy may have a tumor suppressor function, as suggested by the observation that autophagic genes, such as UV radiation resistance-associated gene (*UVRAG*) and *Beclin1*, are frequently deleted in human cancers [27,28]. Furthermore, defective autophagy may lead to increased DNA damage, gene amplification, and chromosomal instability that are associated with tumor progression [29]. On the other hand, when autophagy is activated in poorly vascularized areas of solid tumors, it may help tumor cells to escape nutrient deprivation and promote survival [30,31]. Autophagy is considered a stress adaptive response that avoids cell death by supplying cells with amino acids and ATP. However, it can also contribute to autophagic cell death [31,32] and several studies now support the hypothesis that since autophagy is an important chemoresistance mechanism in multiple malignancies, targetting autophagy may promote cancer cell death [33].

In the present study, we demonstrate that autophagy induced by SAHA impacts mutp53 degradation in MDA-MB-231 breast cancer cells. In this cellular context, the presence of mutp53 confers a slight sensitivity to SAHA, but cell death can be further increased by inhibiting autophagy and, consequently, stabilizing the level of mutp53.

## Results

### SAHA triggers mutant but not wt p53 degradation

**Figure 1:**
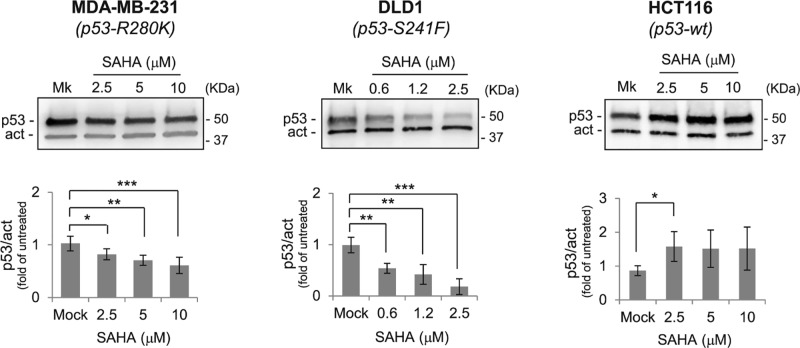
SAHA induced mutp53 degradation and wtp53 increase in cancer cells Representative Western blots showing dose-dependent modulation of mutant and wild-type p53 in MDA-MB-231, DLD1, and HCT116 cells following SAHA treatment. Histograms represent the amount of p53 protein normalized for β-actin and expressed as fold of the untreated sample (Mock, Mk). Data were obtained after chemiluminescence analysis by UVITEC of Western blots from more than three independent experiments; the decrease in mutp53 and the increase in wtp53 in untreated compared with SAHA-treated cells were statistically significant (*P<0.05; **P<0.005; ***P<0.0005) (full-length blots are presented in Supplementary Figure S1).

To investigate the correlation between induction of autophagy by SAHA and p53 degradation, we first determined the modulation of p53 levels induced by SAHA in MDA-MB-231 and DLD1 cells, carrying the mutp53-R280K and the mutp53-R241S mutant proteins, respectively, and in HCT116 wtp53^+/+^ cells (Figure 1). SAHA triggered a significant p53 degradation in both mutp53 carrying cell lines. This effect was more evident in DLD1 cells, where low SAHA concentrations almost completely eliminated mutp53. On the other hand, in HCT116 wtp53^+/+^ cells, the p53 protein was stabilized, indicating that the degrading activity of SAHA is specifically exerted on mutp53 proteins.

### Mutp53 degradation induced by SAHA correlates with autophagy induction in MDA-MB-231 cells

**Figure 2:**
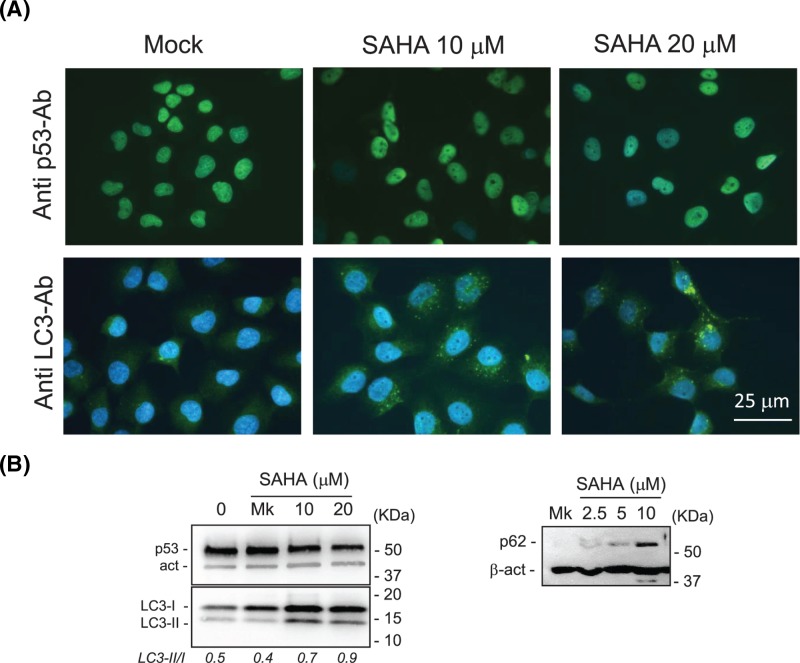
The mutp53 degradation correlates with the appearance of LC-3 positive vacuoles and LC3-II increase in MDA-MB-231 cells (**A**) MDA-MB-231 (mutp53-R280K) were mock- or SAHA-treated for 24 h and fixed for immunostaining with antibodies against p53 or endogenous LC3. Immunofluorescence and DAPI images were taken with a 40× magnification and merged. (**B**) Left panel: representative Western blots showing the increase in LC3-II and the concomitant mutp53 down-modulation in cell extracts obtained after SAHA treatments. The LC3II/I ratio is shown below. Right panel: representative Western blot showing the increase in p62 after SAHA treatment (full-length blots are presented in Supplementay Figure S2). Abbreviation: LC3I/II, microtubule-associated protein light chain 3-I/II.

The mutp53 degradation was also evaluated by immunostaining with an anti-p53 antibody (Figure 2A, upper panels); the green nuclear staining diminished after SAHA exposure of MDA-MB-231 cells, indicating a lower amount of p53 protein in the nuclei.

**Figure 3:**
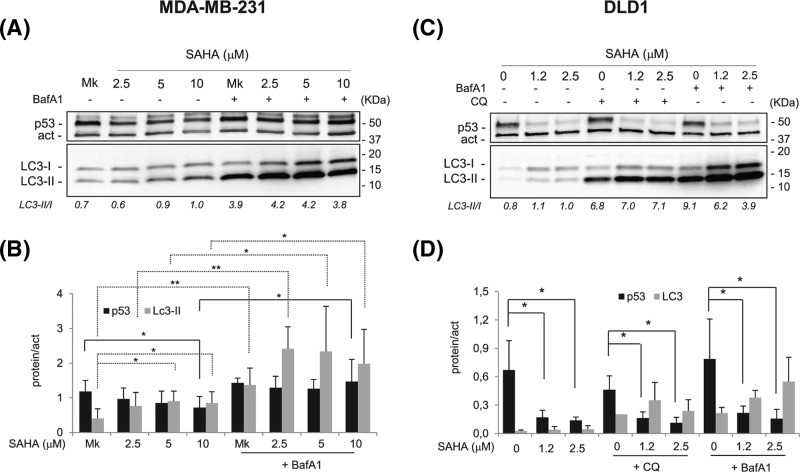
Autophagy inhibition stabilizes mutp53 in MDA-MB-231 but not in DLD1 cells (**A**) Representative Western blots showing the modulation of mutp53 in SAHA-treated MDA-MB-231 cells with or without the addition of BafA1. Cells were treated for 8 h with different SAHA concentrations and 100 nM BafA1 were added for additional 16 h (full-length blots are presented in Supplementary Figure S6). (**B**) Histogram reporting the levels of mutp53 and LC3-II proteins normalized for β-actin, calculated after chemiluminescence analysis by UVITEC of Western blots from three independent experiments (*P<0.05; **P<0.005). (**C**) Representative Western blots showing the down-modulation of mutp53 and the increase in LC3-II in SAHA-treated DLD1 cells with or without the addition of CQ (50 μM) or BafA1 (100 nM). Cells were treated for 8 h with different SAHA concentrations and inhibitors were added for additional 16 h (full-length blots are presented in Supplementary Figure S7). (**D**) Histogram reporting the levels of mutp53 and LC3-II normalized for β-actin, calculated from three independent experiments. Abbreviations: BafA1, Bafilomycin A1; CQ, chloroquine.

In order to correlate the SAHA-induced mutp53 degradation in MDA-MB-231 cells with its autophagic potential, the endogenous Microtubule-associated protein Light Chain 3 (LC3) protein was analyzed by immunofluorescence assays with an antibody against endogenous LC3 protein (Figure 2A). The conversion of the cytosolic LC3-I into LC3-II isoform recruited on the autophagosomal membranes, together with the lysosomal degradation of LC3-II, are considered markers of autophagy progression [34]. Large green vacuoles containing LC3 could be visualized by immunofluorescence in SAHA-treated MDA-MB-231 cells but not in mock cells (Figure 2A), indicating autophagy induction. Western blot analysis confirmed the decrease in mutp53 and its association with the increase in the LC3-II isoform (Figure 2B). However, although the increase in the LC3-II isoform was significant (see histogram in Figure 3B), both the LC3-I and LC3-II isoforms were up-regulated; thus, as a consequence, the ratio of LC3-II/LC3-I only moderately (1.8-fold) increased. The level of p62 protein, also called sequestosome (SQSTM1) and considered an autophagy substrate [34], was also evaluated. The level of p62 decreases when autophagy is induced, while it accumulates when autophagy is inhibited or impaired. In SAHA-treated cells, we found an increase in p62 levels that, together with a moderate increase in the LC3-II/LC3-I ratio, could imply an induction of impaired autophagy that did not allow the complete degradation of this autophagic substrate [35]. The induction of an impaired autophagy could be also deduced by other observations such as an inhibition of free tubulin polymerization and an increase in tubulin acetylation induced by SAHA, in MDA-MB-231 cells (Supplementary Figure S3). The tubulin-microtubule organization within the cells is important for the execution of autophagy, in particular for the fusion between autophagosomes with lysosomes [36]. Furthermore, the hyperacetylation of tubulin may affect the transport of autophagosome to the lysosomes [37]. Thus, we can infer that the effects of SAHA on tubulin (namely the inhibition of tubulin polymerization and tubulin hyperacetylation) can contribute to an impaired autophagy.

The correlation between induction of autophagy and mutp53 degradation induced by SAHA was also investigated in DLD1 cells. As previously shown in Figure 1, SAHA triggered a strong mutp53 degradation in DLD1 cells, also confirmed by the decrease in the green nuclear staining (Supplementary Figure S4A). However, immunofluorescence assays and Western blots showed a weak autophagic potential of SAHA in these cells (Supplementary Figure S4A,B; Supplementary Figure S5).

To monitor the autophagic flux induced by SAHA, the V-ATPase inhibitor Bafilomycin A1 (BafA1) was used as an autophagy inhibitor. BafA1, by changing the pH of lysosomes, impairs the autophagosome–lysosome fusion, but the LC3-II isoforms continue to be produced and accumulated in the membrane of the autophagosome [34]. Indeed, in MDA-MB-231 cells, the addition of BafA1 3 h before the withdrawal of SAHA-treated cells caused a further increase in LC3-positive green vacuoles (Supplementary Figure S6). The modulation of LC3-II and mutp53 following SAHA in the presence of BafA1, as determined by Western blot (Figure 3A,B), confirmed the results obtained by the immunofluorescence assays. Autophagy inhibition, demonstrated by the increase in LC3-II, induced a stabilization of mutp53 (Figure 3A,B). Thus, although in MDA-MB-231 cells SAHA induced an impaired autophagic flux (indicated by p62 accumulation), autophagy inhibition still counteracted mutp53 degradation.

The treatment of DLD1 cells with the autophagy inhibitors BafA1 or Chloroquine (CQ) [34], used as single agents, induced an increase in LC3-II, indicating a block of basal autophagy (Figure 3C,D). The addition of CQ or BafA1 to SAHA-treated cells (16 h before the withdrawal) caused a further increase in LC3-II level, indicating a block of the autophagic flux (Figure 3C,D); however, the combined administration of SAHA and CQ or BafA1 did not affect mutp53 level (Figure 3C,D). Thus, in DLD1 cells the inhibition of autophagy did not affect the level of mutp53, consistent with the slight induction of autophagy by SAHA.

Altogether, these results indicate that mutp53 degradation can be mediated by autophagy in cells that respond to SAHA treatment with autophagy induction, such as MDA-MB-231. However, the degradation of mutp53 appears to be not an autophagy-dependent mechanism in DLD1 cells.

### SAHA-induced cytotoxicity: correlation with autophagy inhibition and p53 level

**Figure 4:**
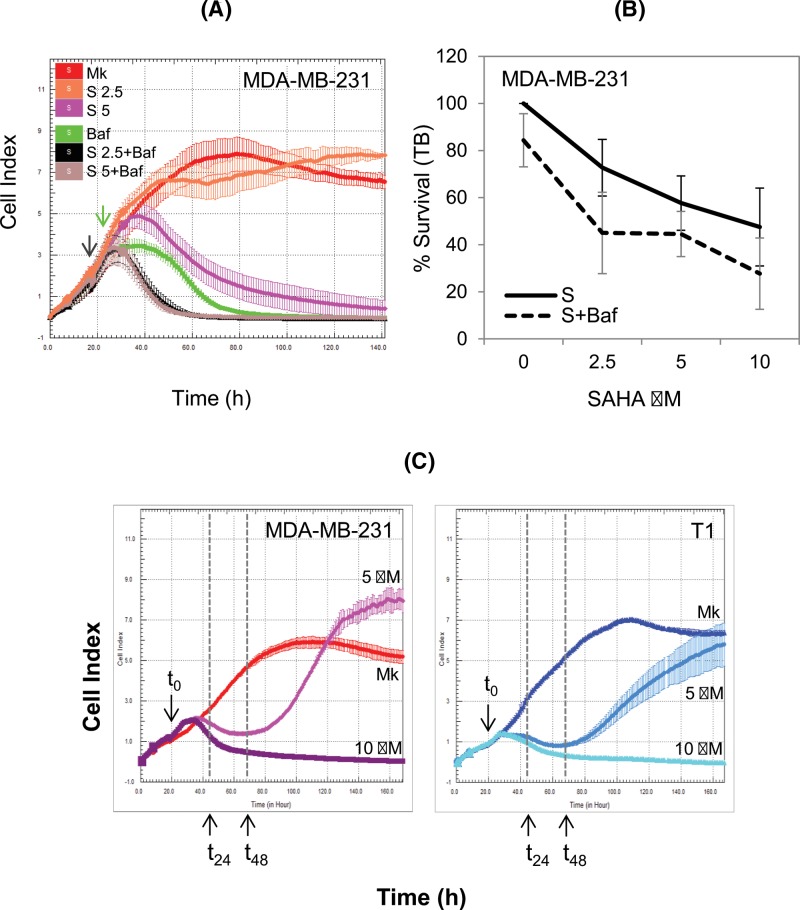
SAHA-induced cytotoxicity: impact of autophagy inhibition and p53 status (**A**) Cell proliferation/survival of SAHA-treated MDA-MB-231 cells measured by xCELLigence RTCA assay. The black and green arrows indicate the time of SAHA and BafA1 (Baf) addition, respectively. (**B**) Cells were treated with different SAHA (S) concentrations with or without the addition of BafA1 (100 nM) for 24 h. The percentage of survival was determined by TB exclusion method as blue/total cells. (**C**) Cell proliferation/survival assays of MDA-MB-231 and T1 SAHA-treated cells measured by xCELLigence RTCA system; the t_0_ arrows indicate the time of SAHA addition.

We next investigated the impact of autophagy and mutp53 degradation on cytotoxicity induced by SAHA. To this aim, autophagy inhibitors (BafA1 or CQ) were added to SAHA-treated cells and cell survival was analyzed by different approaches (see ‘Materials and methods’ section). Using the xCELLigence Real Time Cell Analyzer (RTCA) approach, we registered the Cell Index (CI), a value that takes into account the ability of the cell to establish contacts with the plate, the number of cells, as well as the cells’ morphology. In MDA-MB-231 cells, BafA1 treatment induced a decrease in the CI soon after its addition to the media, and combined treatment of SAHA and BafA1 resulted in a further decrease in the CI (Figure 4A). These data correlated with the Trypan Blue (TB) staining showing that autophagy inhibitors affect SAHA toxicity in MDA-MB-231 cells (Figure 4B), but not in DLD1 cells (Supplementary Figure S8A), in agreement with a less efficient induction of autophagy by SAHA in DLD1 cells. Therefore, the inhibition of autophagy increased SAHA-induced cell death in cells proficient for autophagy induction, such as MDA-MB-231.

We then investigated whether the presence of mutp53 has an impact on SAHA-induced cytotoxicity in our experimental settings. We have previously demonstrated that mutp53-R280K knockdown T1 cells were more sensitive to PRIMA-1 than the parental MDA-MB-231 cells, suggesting a pro-survival function of the mutp53 protein expressed in these cells [13]. Furthermore, it has been shown that SAHA exhibits preferential cytotoxicity in cancer cells carrying various mutp53s [18].

In the case of MDA-MB-231 and T1 cells, cell proliferation, monitored by xCELLigence RTCA, showed a slight p53-dependent effect of SAHA treatment (Figure 4C); the CI profile measured in MDA-MB-231 cells decreased after the addition of SAHA (5 μM), but after approximately 48 h (see the t_48_ arrow in Figure 4C), it started to increase and overcame the CI profile of the mock sample at later times. In T1 cells, a similar CI profile was measured at 5 μM SAHA but it did not overcome the CI of the mock-treated cells. This suggests a better adhesion and proliferation of MDA-MB-231 compared with T1 cells at this SAHA concentration. At a higher concentration (10 μM), SAHA was equally toxic for both cell lines. In the case of DLD1 cells, no difference was observed between mock and treated cells with 5 μM SAHA, while at 10 μM SAHA cells continued to proliferate as the mock cells for approximately 24 h before starting to slowly detach from the plate (evidenced by a decrease in the CI). This indicates that DLD1 are more resistant than MDA-MB-231 and T1 cells to SAHA (Figure 4C and Supplementary Figure S8B). A higher SAHA sensitivity of MDA-MB-231 and T1 cells, compared with DLD1, was also observed by using colony assay (Supplementary Figure S8). The DLD1 cell sensitivity did not correlate with a high mutp53 level, which could confer a high resistance of these cells to SAHA since, as shown above, in DLD1 cells, a strong reduction in the mutant protein level occurred at the low 2.5 μM SAHA concentration, a dose that induced a slight cytotoxicity (∼10%). Human cancer cell lines carrying (MCF7, HCT116 wtp53^+/+^) or lacking (HCT116 wtp53^−/−^) wtp53 showed a comparable sensitivity to MDA-MB-231 and T1 cells in colony assay (Supplementary Figure S8C,D). However, MTT proliferation assay, based on the quantitation of metabolically active cells, showed slightly different results: MCF7 cells were the most resistant at both 24 and 48 h, while HCT116 wtp53^−/−^ cells were the most sensitive 48 h after treatment (Supplementary Figure S8C,D).

Altogether these data clearly indicate that, although cells carrying mutp53 can be more sensitive to SAHA than cells carrying wtp53 (as in the case of MCF7 cells), cell-type and -contexts have to be considered in the interpretation of SAHA cytotoxicity in cancer cell lines.

### SAHA induces apoptosis and G_2_-M cell cycle arrest in a mutp53-independent manner

**Figure 5:**
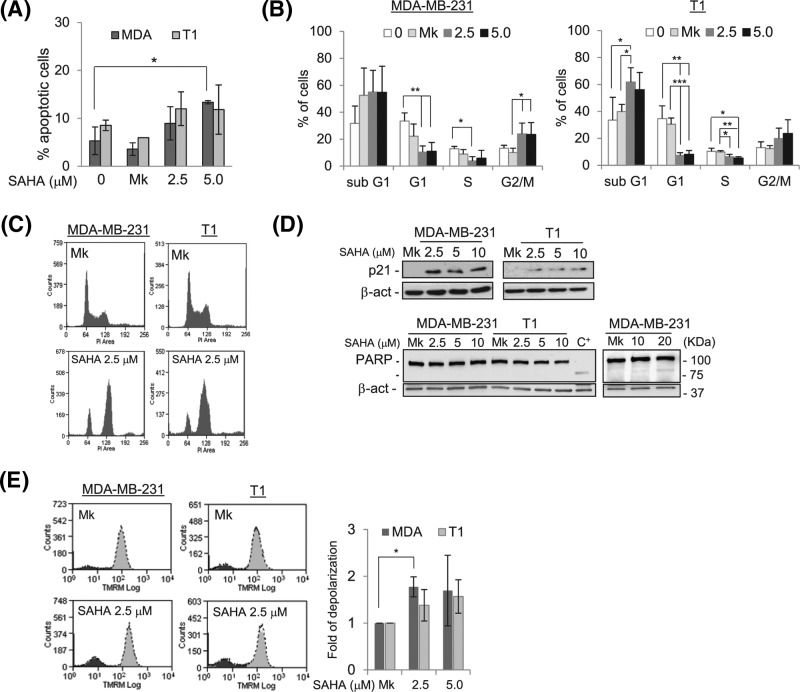
SAHA is not a strong apoptosis inducer but mainly a cytostatic molecule (**A**) Induction of apoptosis after SAHA treatment determined by Annexin/PI assay and (**B**,**C**) cell cycle distribution determined by PI analysis after SAHA treatment. (**D**) Representative Western blots showing the level of p21 and PARP cleavage after SAHA treatment (full-length blots are presented in Supplementary Figure S10). (**E**) Mitochondrial membrane depolarization measured by FACS with TMRM incorporation after SAHA treatment. Histogram below shows the fold of cell membrane depolarization measured in three independent experiments; (*P<0.05; **P<0.005; ***P<0.0005).

We evaluated the death pathways activated by SAHA using several different approaches (see ‘Materials and methods’ section for details). The analysis with Annexin/Propidium Iodide (PI) showed that the percentage of early apoptotic cells increased significantly following SAHA treatment of MDA-MB-231 (Figure 5A) and DLD1 cells (Supplementary Figure S9). The cell distribution in the different phases of the cell cycle, including the sub-G_1_ phase, confirmed a moderate apoptotic potential of SAHA in MDA-MB-231 besides the induction of a significant G_2_/M cell cycle arrest (Figure 5B,C), while in T1 cells a significant increase in sub-G_1_ cell percentage was detected (Figure 5B). Accordingly, the percentage of G_1_ and S phase cells decreased significantly in both cell lines following SAHA treatments. Related to these findings, SAHA exposure led to an up-regulation of p21, but to a negligible poly (ADP-ribose) polymerase (PARP) cleavage, visible only at high SAHA concentrations (Figure 5D). Furthermore, the mitochondrial Tetramethylrhodamine-methyl ester (TMRM) staining incorporation was studied as a further parameter to detect mitochondrial dysfunction that partially correlates with the induction of apoptosis [38]. This red lipophilic fluorescent molecule accumulates within active mitochondria, and in the presence of a mitochondrial damage, the membrane is depolarized causing a fluorescence reduction proportional to the entity of the damage. Flow cytometry (FACS) analysis showed a low but significant variation of the peak of TMRM fluorescence after SAHA treatment in MDA-MB-231 cells (Figure 5E), in agreement with Annexin/PI data.

We obtained comparable results in HCT116 wtp53^+/+^ suggesting that SAHA induced a significant increase in sub-G_1_ and G_2_/M cells, together with a significant decrease in G_1_- and S-phase cell contents (Supplementary Figure S11); however, in these cells, the presence of wtp53 allows SAHA to trigger a more significant extent of apoptosis, measured as the percentage of sub-G_1_ cells and PARP cleavage (Supplementary Figure S11).

In conclusion, these results indicate that SAHA is not a strong inducer of apoptosis and mainly elicits cytostatic effects. Furthermore, the presence of mutp53 did not significantly affect the apoptotic response to SAHA.

## Discussion

Most of the cytotoxic agents currently used in cancer therapy can be rather inefficient against tumors expressing mutp53. It is becoming evident that the treatment with molecules able to eliminate mutp53 proteins, overexpressed in human cancer cells, may allow the removal of the oncogenic functions linked to mutp53 stabilization and, consequently, mutp53-dependent chemoresistance in tumor cells [39–41]. Therefore, there is an urgent need to investigate which mechanisms are involved in the selective degradation of mutp53 in order to develop therapeutic strategies that could overcome chemoresistance.

Recently, a variety of molecules have been characterized for their ability to induce mutp53 degradation through the activation of autophagy. In the present study, we show that autophagy, induced by SAHA, can counteract mutp53 stability by promoting mutp53 degradation; specifically, in MDA-MB-231 cells, SAHA induced a significant level of autophagy (Figures 1 and 2). Although the increase in p62 revealed that the induced autophagy was likely impaired (Figure 2), its inhibition by BafA1 led to the block of the autophagic flux and to mutp53 stabilization (Figure 3). The role of autophagy in mutp53 depletion has been proved in several cell contexts and with different molecules [42]. We have recently demonstrated, for the first time, that PRIMA-1 and GA induce autophagy that selectively elicit mutant but not wt p53 degradation [14,17]. Other studies have shown that molecules such as Zn(II)-compound, capsaicin, and arsenic trioxide induce mutp53 depletion through autophagy [10,11,43]. Finally, it has been reported that, in mouse models, the level of glucose present in the diet affect mutp53 through autophagy [44]. Thus, our results are in support of the role of autophagy in regulating mutp53 stability in cancer cells and reveal a correlation between the autophagic potential of SAHA and its selective ability to perturb mutp53-R280K stability in MDA-MB-231 breast cancer cells.

A different scenario was observed in human colon cancer DLD1 cells, expressing the mutp53-S241F mutant, where we found a weak induction of autophagy and a lack of mutp53 stabilization after autophagy inhibition (Figure 4). Nevertheless, in these cells SAHA triggers a massive and efficient mutp53 degradation, even at low SAHA concentrations (Figure 1). To emphasize this difference, the stability of the two different mutp53 proteins has been explored by transiently transfecting HCT116 wtp53^−/−^ null cells with a plasmid expressing wtp53, mutp53-S241F, or mutp53-R280K proteins [17] and measuring their level following SAHA treatments (Supplementary Figure S9). As expected, in untreated cells, the levels of the two mutp53 proteins remained higher than the wt protein throughout the experiment; however, following SAHA exposure the mutp53-S241F protein appeared more stable than the mutp53-R280K protein (Supplementary Figure S13). This suggests that the massive degradation of the mutp53-S241F protein observed in DLD1 cells after SAHA exposure was not due to intrinsic features of the mutp53, but is more likely related to an alternative degradation pathway rather than autophagy. It has been demonstrated that SAHA, an HDACi, induces the destabilization of the HDAC6–HSP90–mutp53 complex, leading to p53 degradation following its dissociation from this complex [18]. Thus, we can hypothesize that in DLD1 cells, where SAHA did not trigger a significant extent of autophagy, the activity of SAHA on the HDAC6–HSP90 chaperone axis predominated, leading to mutp53 degradation by an autophagy-independent pathway. Therefore, our results indicate that SAHA may act on mutp53 stability by at least two pathways that may be related to the cell context.

An alternative explanation could involve the role of mutp53 in autophagy inhibition. It has been reported that both mutant and wt p53s may inhibit autophagy when localized in the cytoplasm [45]. In this scenario, SAHA, by acting on the HSP90 complex and favoring mutp53 degradation, could remove the inhibition exerted by mutp53 on autophagy. However, in DLD1 cells where low SAHA concentrations are able to nearly eliminate mutp53, a remarkable autophagy induction should be observed (an event that was not observed). Furthermore, in MDA-MB-231 and DLD1 cells, the mutp53 proteins accumulate in the nucleus [13], making the mutp53 inhibition of autophagy difficult to occur. On the other hand, a mutp53 degradation activity of SAHA-induced autophagy must exist in MDA-MB-231 cells, since autophagy inhibition by BafA1 resulted in mutp53 protein stabilization (Figure 3). Thus, although interplay between these mutp53 degradation pathways—autophagy and HDAC6–HSP90 chaperone axis—may occur, it still remains to be elucidated, and we hypothesize that autophagy induced by SAHA can lead to the degradation of mutp53 in a cell-context dependent manner.

Regardless of the mechanism, an important consequence of mutp53 degradation is the elimination of the pro-oncogenic functions of mutp53 itself which are often responsible for cell survival and/or chemoresistance. According to Li et al. [18], SAHA showed preferential cytotoxicity for cancer cells carrying mutp53, with respect to null or wtp53 expressing cells. In the cell lines analyzed, this observation was partially true: DLD1 cells, carrying the mutp53-S241F, showed less SAHA sensitivity than MDA-MB-231 cells, carrying the mutp53-R280K, in the colony formation assay and the xCELLigence real time cell proliferation assay (Figure 5); yet they showed comparable sensitivity to wtp53-carrying HCT116 cells in the MTT assay (Supplementary Figure S8C). In a previous study it was reported that the DLD1 and ES2 cell lines [18] show a remarkable difference in SAHA sensitivity, even though they carry the same mutp53-S241F protein [46,47], emphasizing the important contribution of cell context to SAHA cytotoxicity. Moreover, our results keep with the significant different SAHA sensitivity of three breast cancer cell lines (including MDA-MB-231) carrying different mutp53 proteins [48]. Nevertheless, in our study, wtp53-carrying MCF7 cells were indeed the most resistant amongst all cell types tested (Supplementary Figure S6).

The T1 cell line, lacking mutp53-R280K, had a slightly greater sensitivity than the parental MDA-MB-231 cells, depending on the SAHA concentration and the assay used (Figure 5C,D). In another study, SAHA-induced cytotoxicity was determined in MDA-MB-231 cells and their derivative p53 knockdown cells by a Tet-inducible shp53RNAi [18]. Li et al. [18] reported that SAHA was less cytotoxic in cells lacking mutp53, but was still able to induce cell death. This may be attributed to an incomplete p53 elimination by Tet-inducible and/or to p53-independent SAHA effects. In our study, T1 cells are stably transfected with a pSUPERp53 vector and completely deficient in mutp53 protein [13] allowing us to hypothesize that an alternative mutp53-independent SAHA activity may play a role in cell survival. Of note, the amount of mutp53 protein did not correlate with SAHA sensitivity in the two mutp53 carrying cell lines analyzed in our study. In fact, DLD1 cells were more resistant than MDA-MB-231, but in DLD1 cells a strong reduction in the mutp53 level occurred even at the lowest SAHA concentration which induced a very weak cytotoxicity (approximately 10%). In agreement with our data, shRNA-mediated knockdown of p53 in Ba/F3 p210 and Ba/F3 T3151 chronic myelogenous leukemia cells did not affect the sensitivity to SAHA [49]. Therefore, our results indicated that, at least in our panel of cell lines, SAHA sensitivity can be marginally related to the presence of mutp53.

An important issue to be addressed is the role of SAHA-induced autophagy on cell death/survival. If SAHA is able to trigger autophagy that, in turn, can weaken the pro-oncogenic functions of mutp53 by promoting its elimination, the inhibition of autophagy should stabilize mutp53 and decrease cell sensitivity. This has been shown in several cases in which the chemical or genetic inhibition of autophagy resulted in mutp53 stabilization and reduced cell death [10,11,17,44]. Here, in order to investigate the role of SAHA-induced autophagy on cell death, we co-treated cells with SAHA and autophagy inhibitors and monitored cell survival and proliferation (Figure 5). The inhibition of autophagy *per se* was toxic for MDA-MB-231, but not for DLD1 cells. Following a combined treatment with SAHA and autophagy inhibitors, MDA-MB-231, but not DLD1 cells, increased their sensitivity indicating that the inhibition of autophagy increased SAHA-induced cell death in cells proficient for autophagy induction. Thus, the inhibition of autophagy did stabilize mutp53 but it did not reduce cell death, as hypothesized above. This indicates that autophagy induced by SAHA protects MDA-MB-231 cells from death, underlining its pro-survival activity.

To investigate the cell death pathway induced by SAHA, the induction of apoptosis by SAHA was studied (Figure 5). In agreement with what is reported in the literature [50], we found a moderate apoptotic activation following SAHA. Instead, we observed a significant G_2_/M cell cycle arrest, particularly in MDA-MB-231 cells (Figure 5). Consistent with these findings, SAHA exposure led to an up-regulation of p21, yet to negligible PARP cleavage (Figure 5D). Indeed, it has been demonstrated that p21 is not only a central regulator of the G_1_/S cell cycle phase and a transducer of stress stimuli in the DNA damage response pathway, but also an important player at the G_2_/M transition and in mitotic progression [51,52]. Interestingly, the CI profiles measured by xCELLigence RTCA for MDA-MB-231 and T1 cell lines following treatment with 5 μM SAHA showed an initial CI decline followed by a recovery (Figure 5C), a CI profile compatible with a global cellular morphological response to antimitotic agents, as reported [53]. Altogether our results confirm that SAHA is not a strong apoptosis inducer but acts mainly by promoting cytostatic events.

The role of apoptosis and autophagy in SAHA-induced cell death has been thoroughly investigated [23,25]. Gammoh et al. [25] found that autophagy inhibition accelerated apoptotic cell death in resistant glioblastoma cells T98G, but in the absence of apoptosis (through co-treatment with a caspase inhibitor), the suppression of autophagy also increased cell death, demonstrating that autophagy has a protective role also toward SAHA-induced non-apoptotic cell death. Thus, SAHA-induced autophagy appears to act as a pro-survival mechanism that counteracts the cytotoxic activity of SAHA by delaying the onset of apoptosis [25]. Although the contribution of autophagy to cell death remains controversial and, most likely, context-dependent, these results indicate that targetting autophagy during treatment with SAHA may augment its therapeutic effects and can have important clinical implications in treating cancers with apoptotic defects. Nevertheless, although SAHA protects from apoptotic and non-apoptotic cell deaths, the role of autophagy in non-apoptotic cell death is yet to be clarified [54]. Since cross-talk between autophagy and apoptosis has been demonstrated [32], we cannot exclude that the chemical inhibition of autophagy would trigger an apoptotic cell death in a mutp53-independent manner. It has been shown that uterine sarcoma p53-deficient cells undergo autophagy when treated with SAHA, whereas cells expressing a functional wtp53 activate apoptosis under the same stimulus [55]. These results identified p53 as a molecular switch that directs cells to autophagy or apoptosis depending on the p53 status [55]. In keeping with this assumption, we also observed a clear apoptosis induction in wtp53-carrying HCT116 cells following SAHA treatment as evidenced by sub-G_1_ cell content and PARP cleavage (Supplementary Figure S11; Supplementary Figure S12) and a lack of significant induction of autophagy (data not shown).

Here, we demonstrated that the autophagic potential of SAHA correlates with specific degradation of mutp53 in human cancer cells. However, SAHA is able to induce different mutp53 degradation pathways that are likely context-dependent, and their cross-talk deserves further investigations. This should also be investigated in relation to features of specific mutp53 proteins. All together these results support the pro-survival role of SAHA-induced autophagy, despite its contribution to mutp53 degradation. The mutp53 stabilization occurring after autophagy inhibition in MDA-MB-231 cells is associated with a decreased cell survival, suggesting that, in this cellular context, the presence of mutp53 does not induce chemoresistance but, on the contrary, supports cell sensitivity to SAHA. Thus, drug combinations with SAHA and autophagy inhibitors might give a therapeutic advantage for the treatment of malignancies, regardless of the presence of mutp53. Interestingly, several clinical trials using SAHA or other HDACi are in progress in different types of tumors [56,57]. Amongst these, SAHA and hydroxychloroquine are undergoing clinical trials in patients with solid and hematopoietic tumors [58–60]. A better understanding of the role of autophagy induced by SAHA-like molecules in chemoresistance in specific tumor subsets will help to stratify cancer patients as well as to develop alternative therapeutic options.

## Materials and methods

### Cell culture and drug treatments

MDA-MB-231 cell line (human breast carcinoma) carrying the *TP53* R280K mutation [61] was authenticated by DNA (STR) profiling (DSMZ, Braunschweig, Germany). The mutp53 knocked-down T1 cell line was obtained as described [13]. The human colon carcinoma HCT116 wtp53^+/+^ and HCT116 wtp53^−/-^ cells (wt, wild-type) were obtained by Dr. B. Vogelstein (The Johns Hopkins Kimmel Cancer Center, Baltimore, MD). MCF7 (expressing wt p53 protein) and DLD1 cells (human colorectal adenocarcinoma) (expressing the mutp53-S241F protein) were obtained by the Interlab Cell Line Collection (ICLC Genova, Italy). Cells were grown in D-MEM (MDA-MB-231, T1) or RPMI (HCT116, DLD1, MCF7) (Gibco Invitrogen, Milano, Italy) containing 5% (MDA-MB-231, T1) and 10% (HCT116, DLD-1, MCF7) FBS (Euroclone, Milano, Italy) and maintained at 37°C in 5% CO_2_ at 100% humidity. Suberoylanilide hydroxamic acid (SAHA) (SML0061, Sigma–Aldrich, Milano, Italy) and V-ATPase inhibitor BafA1 (B1793, Sigma–Aldrich, Milano, Italy) were dissolved in DMSO at a concentration of 50 mM and 16 μM, respectively. Chloroquine (CQ) (C6628, Sigma–Aldrich, Milano, Italy) was dissolved in PBS^−^ at a concentration of 100 mM. Autophagy inhibition was carried out by adding Bafilomycin A1 (BafA1) (100 nM) for 3 or 16 h to SAHA treatment or by a combined treatment of SAHA with CQ (50 μM). Working solutions were prepared by appropriate dilutions in PBS^−^.

### Immunocytochemistry

Cells were seeded on coverslips and treated for 24 h. At the end of treatments, slides were washed with PBS^−^, fixed in 1:1 methanol:acetone and incubated with anti-LC3 (microtubule-associated protein light chain 3) antibody (L8918, Sigma–Aldrich, Milano, Italy) or anti p53 DO-1 antibody (Santa Cruz Biotechnology Inc., Milano, Italy) for 1 h at 37°C, followed by incubation with an anti-rabbit FITC–conjugated or an anti-mouse FITC–conjugated antibody (Sigma–Aldrich, Milano, Italy), respectively. Slides were then counterstained by DAPI. Micrographs were taken using an epifluorescence Provis AX70 microscope (Olympus, Tokyo, Japan) and Cytovision software (Applied Imaging Corp., Santa Clara, CA, U.S.A.).

### Protein extraction and Western blot analysis

Adherent cells were harvested and washed with PBS^−^. Cell lysis was performed in buffer containing 50 mM Tris/HCl pH 7.5, 150 mM NaCl, 1% NP-40, 10% glycerol, 10 mM EDTA, 1 mM DTT, and protease inhibitors (0.5 mM PMSF, 1 mg/ml leupeptin, 2 mg/ml aprotinin, 1 mg/ml pepstatin A). Cell lysates were incubated for 30 min at 4°C and centrifuged at 14000 rpm at 4°C for 5 min. Supernatants were collected and protein concentration was determined using the BCA assay (Bio-Rad, Milano, Italy). Usually 10–20 μg of total proteins were resolved on 7.5–12% SDS/PAGE or Mini protean TGX precast gels (Bio-Rad) and transferred to nitrocellulose Hybond-C Extra membrane (Amersham, GE Healthcare, U.K.). Membranes were blocked with 5% non-fat dry milk in 0.1% Tween-20 in PBS for 1 h, then incubated for 1 h at room temperature or overnight at 4°C with the appropriate primary antibody. The following antibodies were employed: anti-p53 (DO-1, sc-126, Santa Cruz Biotechnology), anti-LC3 I/II (NB100-2220, Novus Biologicals, U.S.A.), anti-p62 (sc-25575, Santa Cruz Biotechnology), anti-poly (ADP-ribose) polymerase (PARP) (PA5-16561, Thermo Fisher Scientific), anti β-actin (AC-74, Sigma–Aldrich, Milano, Italy), secondary anti-mouse IgG peroxidase conjugate (A9044, Sigma–Aldrich, Milano, Italy) and secondary anti-rabbit peroxidase conjugate (A9169, Sigma–Aldrich, Milano, Italy). Detection was carried out with ECL FAST PICO (ECL-1002, Immunological Sciences, Roma, Italy). Chemiluminescence was analyzed by Alliance LD, UVITEC Cambridge (Cambridge, U.K.).

### Trypan Blue assay

Trypan Blue (TB) exclusion method was used to determine cell survival following SAHA exposure in the presence of BafA1. Attached cells were trypsinized and cell suspension was mixed with an equal volume of TB (Sigma–Aldrich, Italy). Approximately 200 cells/point were scored using a hemocytometer and the percentage of cell survival or cell death was determined by counting white/total or blue/total cells, respectively.

### Real-time monitoring of cells proliferation by xCELLigence

For cell proliferation assays, 50 μl of medium was added to a tissue culture plate (E-plate) of the xCELLigence Real Time Cell Analyzer (RTCA) Instrument® (Roche) to obtain background readings, followed by the addition of 50 μl of cell suspension at different densities depending on cell type. The E-plates containing the cells were incubated at room temperature for 30 min and then placed on the reader in the incubator. Cell spreading and proliferation are expressed as an arbitrary unit called Cell index (CI), which is the result of the number of cells, cell morphology and cellular adhesion to the plates. Impedance-based CI was measured with time intervals varying from 5 min to 1 h. After approximately 24 h, cells were treated with different concentrations of the drug and monitored every 30 min for the designed period of time.

### Apoptosis determination

Apoptotic cells were analyzed using Annexin V-FITC/PI double staining method and by the determination of the percentage of sub-G_1_ cells. Cells were harvested by trypsinization 24 h after treatment, pooled with the floating cells, centrifuged for 10 min at 1000 rpm and re-suspended in cold PBS at the concentration of 10^6^ cells/ml. For AnnexinV/propidium iodide (PI) analysis aliquots of 5 × 10^5^ cells were processed using Annexin V-FITC Apoptosis detection kit (Calbiochem-Merck, Milan, Italy) according to the manufacturer’s instructions. The percentage of cells undergoing early (FITC positive, PI negative) and late stage (FITC positive, PI positive) apoptosis was assessed by flow cytometry via dual-color analysis on 10000 gated cells using a CyAn ADP cytometer (Beckman Coulter, Brea, CA, U.S.A.). For the analysis of the sub-G_1_ fraction and cell cycle distribution, aliquots of 5 × 10^5^ cells were re-suspended in 1 ml of cold PBS containing 0.1% Triton X-100 and 50 μg/ml PI (Sigma–-Aldrich, Italy) and analyzed by flow cytometry on the CyAn ADP cytometer (Beckman Coulter, Mountain View, CA, U.S.A.) equipped with three laser lamps. The percentage of cells in different phases of the cell cycle as well as the sub-G_1_ population were determined using the inbuilt software (BD CellQuest software).

The mitochondrial membrane depolarization was measured by Tetramethylrhodamine-methyl ester (TMRM) (Invitrogen, Milano, Italy) staining. TMRM is a colorless dye that is readily sequestered by mitochondrial lipid membrane and converted into a bright red-orange fluorescent molecule. The molecule, which is excited at 488 nm, displays a decrease in fluorescence when the polarization of the mitochondrial membrane is decreased by treatments, that make it leaky, an early step in apoptosis activation. The proportion of cells that display reduced TMRM fluorescence is directly proportional to the entity of the depolarization, indicated in figures as a mitochondrial membrane depolarization, and calculated as the percentage of the total.

### Statistical analysis

Statistical significance of the observed differences amongst the experimental groups was calculated using a two-tailed unpaired *t*test. Values were presented as mean ± S.D. of at least three independent experiments or as indicated. A *P*-value of less than 0.05 was considered to be statistically significant. In the figures, *, ** and *** indicate statistical significance at *P*<: 0.05, 0.005, and 0.0005, respectively. The statistical calculations were performed with GraphPad Prism 6.0 for Windows (GraphPad Software, La Jolla, CA, U.S.A.).

## Supporting information

**Supplementary Figure:** Western blot analysis and chemiluminescence detection with UVITEC For the Western blot analysis with the UVITEC system, we set up a procedure to perform the incubation with up to 4 different antibodies on the same membrane. After protein transfer and staining with the Ponceau Red of the membrane, an image with molecular weight marker is acquired **(A)**. The membrane is then cut in three parts, according to the molecular weight marker positions, in order to perform incubations with different antibodies (Ab) (PARP, p53 and β-actin, LC3). Following the incubation with each Ab (p53 and β-act in this figure), the chemiluminescence is acquired for protein quantification **(B)**. To get a precise report of all protein bands, after the acquisition of the chemiluminescence signal of each antibody, the U, M and B membrane portions are joined and an image of the whole membrane, merged with the molecular weight marker, is taken **(C)**.

**Supplementary Figure S1:** Full-length blots of proteins illustrated in Fig 1. For each cell lines (as indicated), the membrane loaded with protein extracts of cells treated with different SAHA concentrations were processed as described in Suppl Informations. The membranes M were first hybridized with the anti-p53 Ab (DO1) and then with the anti-β-actin Ab. After primary antibodies hybridizations, the membranes were hybridized with the common secondary Ab and the chemiluminescence from p53 and β-actin was analyzed in the same UVITEC acquisition. The membranes B were hybridized with the anti-LC3 Ab, followed by hybridization with the secondary Ab and chemiluminescence was analyzed by UVITEC. After the signal acquisition for each antibody, the M and B membranes were joined and an image of the whole membrane, merged with the molecular weight marker, was taken. The orange squares outline the cropped areas reported in Fig 1.

**Supplementary Figure S2:** Full-length blots of proteins illustrated in Fig 2B. **A)** The membrane loaded with protein extracts of MDA-MB-231 cells treated with different SAHA concentrations were processed as described in Suppl Informations. The membranes M was first hybridized with the anti-p53 Ab (DO1) and then with the anti-β-actin Ab. After primary antibodies hybridizations, the membrane was hybridized with the common secondary Ab and the chemiluminescence from p53 and β-actin was analyzed in the same UVITEC acquisition. The membranes B was hybridized with the anti-LC3 Ab, followed by hybridization with the secondary Ab and chemiluminescence was analyzed by UVITEC. After the signal acquisition for each antibody, the M and B membranes were joined and an image of the whole membrane, merged with the molecular weight marker, was taken. The orange squares outline the cropped areas reported in Fig 2B, right panel; **B)** The membrane loaded with protein extracts of MDA-MB-231 cells treated with different SAHA concentrations were first hybridized with the anti-p62 Ab (Santa Cruz Biotech., sc-25575) and then with the anti-β-actin Ab. Chemiluminescence of protein bands was detected by autoradiographic film exposure. The orange square outlines the cropped area reported in Fig 2B, left panel.

**Supplementary Figure S3:** A) In vitro tubulin polymerization assay. The effect of SAHA on the in vitro assembly of tubulin microtubules was evaluated using a fluorescence-based microtubule polymerization assay kit (Cytoskeleton Inc., Denver, USA), according to the manufacturerߣs protocol. Briefly, 2mg/ml porcine brain tubulin (>99% pure with minimal contamination of microtubule-associated proteins) was incubated with tubulin buffer, cushion buffer, GTP without or with 2.5-5 μM SAHA in a pre-warmed half area 96-well plate (Corning Costar) and the reaction was initiated by the addition of tubulin. Three μM Taxol and 1.5 μM Vincristine were used for enhancing or suppressing tubulin polymerisation, respectively (positive controls). The plate was incubated at 37 °C in a fluorescence microplate reader (Mithras LB 940, Berthold Technologies), and microtubule assembly was monitored by measuring the increase in fluorescence due to the incorporation of a fluorescence reporter into microtubules as polymerization proceeds. Fluorescence reading (γ_ex_, 355 nm; γ_em_, 460nm) was done every 5 minutes for 60 min, with 5 sec shaking every cycle. B) Increase of acetylated tubulin in cell extracts from SAHA-treated MDA-MB-231 cells as shown by western blot analysis.

**Supplementary Figure S4:** Mutp53 degradation and LC3-II increase induced by SAHA in DLD1 cells. **A)** DLD1 (p53-S241F) were mock- or SAHA-treated for 24 h and fixed for immunostaining with antibodies against p53 or endogenous LC3. Immunofluorescence and DAPI images were taken with a 40x magnification and merged; **B)** Representative western blots showing the mutp53 down-modulation and the LC3-II increase in cell extracts from SAHA-treated cells. The histogram below shows the level of LC3-II normalized for β-actin obtained after chemiluminescence analysis by UVITEC of western blots from more than three independent experiments (*p<0.05).

**Supplementary Figure S5:** Full-length blots of proteins illustrated in Suppl Fig S4. The membrane were processed as described in Suppl Informations. **A)** The membrane M was hybridized with the anti-p53 Ab (DO1), the secondary Ab and the chemiluminescence from p53 was acquired by UVITEC (M1). The same membrane was then hybridized with the anti-β-actin Ab and after incubation with the secondary Ab, the chemiluminescence was again acquired with UVITEC (M2). The membranes B was hybridized with the anti-LC3 Ab, followed by hybridization with the secondary Ab and chemiluminescence was acquired by UVITEC (B). For each Ab, the first chemiluminescence acquisition is used for protein quantification and is shown on the manuscript figure. The orange squares outline the cropped areas reported in Suppl Fig S4. The image of the whole membrane (containing cell extracts from another experiment), merged with the molecular weight marker, was taken; **B)** The membrane were first hybridized with the anti-LC3 Ab (Santa Cruz Biotech., sc-25575) and then with the β-actin Ab. After hybridization with the respective secondary Abs, chemiluminescence of protein bands was detected by autoradiographic film exposure. The orange squares outline the cropped areas reported in Suppl. Fig S4.

**Supplementary Figure S6:** Autophagy inhibition stabilizes mutp53 in MDA-MB-231 cells **A)** MDA-MB-231 (p53-R280K) cells were mock or SAHA-treated for 24 h and fixed for immunostaining with antibodies against endogenous LC3. BafA1 was added in mock- and SAHA-treated cells 3 h before the withdrawal. Immunofluorescence and DAPI images were taken with a 40x magnification and merged. The addition of BafA1 3 h before the withdrawal of SAHA-treated cells caused a further increase of LC3-positive green vacuoles (compare right upper and right lower panels); **B)** *Full-length blots of proteins illustrated in Fig 3A.* The membrane loaded with protein extracts of MDA-MB-231 cells treated with different SAHA concentrations, with or without BafA1, were processed as described in Suppl Informations. The membranes M was first hybridized with the anti-p53 Ab (DO1) and then with the anti-β-actin Ab. After primary antibodies hybridizations, the membranes was hybridized with the common secondary Ab and the chemiluminescence from p53 and β-actin was analyzed in the same UVITEC acquisition. The membrane B was hybridized with the anti-LC3 Ab, followed by hybridization with the secondary Ab and chemiluminescence was analyzed by UVITEC. The orange squares outline the cropped areas reported in Fig 3A.

**Supplementary Figure S7:** Full-length blots of proteins illustrated in Fig. 3C. The membrane were processed as described in Suppl Informations. The membrane M was hybridized with the anti-p53 Ab (DO1), the secondary Ab and the chemiluminescence from p53 was acquired by UVITEC. The same membrane was then hybridized with the anti-β-actin Ab and after incubation with the secondary Ab, the chemiluminescence was again acquired with UVITEC. The membranes B was hybridized with the anti-LC3 Ab, followed by hybridization with the secondary Ab and chemiluminescence was acquired by UVITEC (B). For each Ab, the first chemiluminescence acquisition is used for protein quantification and is shown on the manuscript figure. The orange squares outline the cropped areas reported in Fig 3C. After the signal acquisition for each antibody, the M and B membranes were joined and an image of the whole membrane, merged with the molecular weight marker, was taken.

**Supplementary Figure S8:** A) Trypan blue assay in DLD1 Cells were treated with different SAHA (S) concentrations with or without the addition of BafA1 (100 μM) or CQ (50 mM) for 24 h. The percentage of survival was determined by trypan blue exclusion method as blue/total cells. B) xCELLigence RTCA assay in DLD1 Cell proliferation/survival assays of SAHA-treated DLD1 cells measured by xCELLigence system; the t_0_ arrows indicate the time of SAHA addition. SAHA-induced cytotoxicity measured in MDA-MB-231, T1, DLD1 MCF7, HCT116 wtp53^+/+^ and HCT116 wtp53^-/-^ cell lines by colony forming and MTT assays. For colony forming assay cells were seeded at a density of 400 cells and allowed to growth for 24 h. Cells were treated by adding the drug directly in the medium at the indicated concentrations. After 24 h of treatment, the medium was removed and replaced with complete fresh medium. After 8-10 days, the colonies were counted. For MTT, cells were seeded in 96-well and treated with different SAHA concentrations in triplicates. The percentage of viable cells was determined after 24h and 48h of treatment. The average and the standard deviations of at least three independent experiments are reported.

**Supplementary Figure S9:** SAHA induced apoptosis and G2/M arrest in DLD1 cells. **A)** Induction of apoptosis after SAHA treatment determined by Annexin/PI assay (*p<0.05); **B)** Representative western blots showing the level of p21 and PARP cleavage after SAHA treatment. (Full-length blots are presented in Supplementary Fig. S9); **C)** Representative PI cell cycle profiles of Mock and SAHA-treated DLD1 cells.

**Supplementary Figure S10:** Full-length blots of proteins illustrated in Fig 5D and Suppl Fig S9. **A)** For each cell lines (as indicated), the membrane loaded with protein extracts of cells treated with different SAHA concentrations were first hybridized with the anti-p21 Ab and then with the anti-β-actin Ab. Chemiluminescence of protein bands was detected by autoradiographic film exposure; **B)** The membranes were processed as described in Suppl Inf: the upper membrane (U) was incubated with PARP Ab, the M membrane was incubated with p53 and β-actin Abs. Following the incubations with the respective secondary Abs, chemiluminescence was acquired. The whole membrane, merged with the molecular weight marker is reported in Suppl Informations figure; **C)** The membrane is the same as in Fig. S2A. After p53 and β-actin Abs ibridization, the membrane was incubated with PARP Ab and overexposed to see the faint band of cleaved PARP. For the whole membrane with MW marker see Fig. S2A; **D)** Membranes loaded with protein extracts (as indicated) were processed as above: the upper membranes (U) were incubated with PARP Ab, the M membranes were incubated with p53 and β-actin Abs. Following the incubations with the respective secondary Abs, chemiluminescence was acquired. The U and M membranes were joined and an image of the whole membrane, merged with the molecular weight marker, was taken. The orange squares outlines the cropped areas reported in Fig 5D.

**Supplementary Figure S11:** SAHA induced apoptosis and G_2_/M arrest in wtp53^+/+^ and wtp53^-/-^ HCT116 cells. **A)** Percentage of subG1 cells and cell cycle distribution determined by PI analysis in SAHA-treated HCT116 wtp53^+/+^ and HCT116 wtp53^-/-^ cells. For HCT116 wtp53^-/-^ cells, only one PI experiment could be analyzed; **B)** Representative PI cell cycle profiles of Mock and SAHA-treated HCT116 cells; **C)** Representative western blots showing the level of p21 and PARP cleavage after SAHA treatment. (*p<0.05; **p<0.005). Full-length blots of proteins are presented in Fig S12.

**Supplementary Figure S12:** Full-length blots of proteins illustrated in Fig S11. **A)** Membranes loaded with cell extracts of HCT116 wtp53^+/+^ and HCT116 wtp53^-/-^ treated with different SAHA concentrations (as reported on the autoradiographic film) were first hybridized with p21 Ab and then with the anti-β-actin Ab. The protein molecular weights are shown on the film. The orange squares outlines the cropped areas reported in Fig S11,C; **B)** The membrane loaded with cell extracts of HCT116 wtp53^+/+^ and HCT116 wtp53^-/-^ treated with different SAHA concentrations were processed as described in Suppl Informations. The membrane U was hybridized with the anti-PARP Ab, followed by hybridization with the secondary Ab; chemiluminescence was analyzed by UVITEC. The membrane M was first hybridized with the anti-p53 Ab (DO1) and then with the anti-β-actin Ab. After primary antibodies, the membrane was hybridized with the common secondary Ab and the chemiluminescence from P53 and β-actin was analyzed in the same UVITEC acquisition. The membrane B was hybridized with the anti-LC3 Ab and chemiluminescence was analyzed by UVITEC. The orange squares outlines the cropped areas reported in Fig S11,C; **C)** After the signal acquisition for each antibody, the U, M and B membranes were joined and an image of the whole membrane, merged with the molecular weight marker, was taken.

**Supplementary Figure S13:** Modulation of wtp53, p53-S241F and p53-R280K proteins in transiently transfected HCT116 wtp53^-/-^ cells following SAHA treatment. **A)** HCT116 wtp53^-/-^ cells were transiently transfected with plasmid expressing wtp53, p53-S241F and p53-R280K proteins as previously described (Foggetti et al., 2017). After 24 h (T_0_), cells were treated with SAHA for 8 h and 24 h. Cells were collected and cell extracts prepared. Western blot showing the level of p53s in transfected cells treated with SAHA is reported, together with the p53/β-actin ratio calculated by UVITEC analysis. (Full-length blots of proteins are presented in Fig S14). B) To better highlight the modulation of wtp53, p53-S241F and p53-R280K proteins after treatment with different SAHA concentrations, a histogram was constructed by normalizing each p53/β-actin value reported above for the p53/β-actin value calculated at T_0_.

**Supplementary Figure S14:** Full-length blots of proteins illustrated in Fig S13. Membranes (M1 and M2) loaded with cell extracts of HCT116 wtp53^-/-^ cells transiently transfected with plasmid expressing wtp53, p53-S241F and p53-R280K proteins were first hybridized with the anti-p53 Ab (DO1) and the chemiluminescence was acquired with UVITEC. Then the same membranes were incubated with the anti-β-actin Ab and after incubation with the secondary Ab, the chemiluminescence for β-actin was acquired. The orange squares outlines the cropped areas reported in Fig S13.

**Supplementary Figure S15:** Mutp53 and LC3-II protein level in MDA-MB-231 cells treated with 100nM Rapamycin, and SAHA, alone and in combination. **A)** Representative western blots showing the mutp53 down-modulation and the LC3-II increase in cell extracts from SAHA-treated cells. The combined SAHA+ rapamycin treatment did not affect the level of mutp53 in comparison with SAHA alone; **B)** To better highlight the results, the histogram shows the level of LC3-II and p53 normalized for β-actin obtained after chemiluminescence analysis by UVITEC of western blots.

## Abbreviations

BafA1: Bafilomycin A1
CI: cell index
CQ: chloroquine
GA: gambogic acid
HDACi: histone deacetylases inhibitor
HSP90: heat shock protein 90
LC3-I/II: microtubule-associated protein light chain 3-I/II
mutp53: mutant p53
PARP: poly (ADP-ribose) polymerase
PI: propidium iodide
PRIMA-1: P53 re-activation and induction of massive apoptosis
RTCA: Real Time Cell Analyzer
SAHA: suberoylanilide hydroxamic acid
TB: Trypan Blue
TMRM: tetramethylrhodamine-methyl ester
wt: wild-type
