# Supplementary material for: Autophagy induced by SAHA affects mutant P53 degradation and cancer cell survival
Source: Biosci Rep. 2019 Feb 19;39(2):BSR20181345. doi: 10.1042/BSR20181345 (PMC6379511; doi:10.1042/BSR20181345)
Supplement: Supplementary file 1 [file bsr-39-bsr20181345_Supp1.pdf]

# Supplementary Figures and Informations

## AUTOPHAGY INDUCED BY SAHA AFFECTS MUTANT P53 DEGRADATION AND CANCER CELL SURVIVAL

Giorgia Foggetti[1§], Laura Ottaggio[1], Debora Russo[1^], Carlotta Mazzitelli[1], Paola Monti[1], Paolo Degan[1], Mariangela Miele[1], Gilberto Fronza[#1], and Paola Menichini[#1*].

[1]Mutagenesis and Cancer Prevention Unit, Ospedale Policlinico San Martino, 16132, Genoa, Italy;
[§]Present address: Yale Cancer Center, Yale University School of Medicine, New Haven, Connecticut.
[^]Present address: D3-Pharma Chemistry Line, Istituto Italiano di Tecnologia (IIT), Via Morego 30, 16163, Genoa, Italy

[#] equally contributed to this work

**Corresponding author**: Dr. Paola Menichini
Mutagenesis and Cancer Prevention Unit
Ospedale Policlinico San Martino
L.go R. Benzi, 10
Genoa, Italy
Tel +39.010.555.8225
Fax +39.010.555.8237
Email: paola.menichini@hsanmartino.it

# Supplementary Informations

*Western blot analysis and chemiluminescence detection with UVITEC*

For the Western blot analysis with the UVITEC system, we set up a procedure to perform the incubation with up to 4 different antibodies on the same membrane. After protein transfer and staining with the Ponceau Red of the membrane, an image with molecular weight marker is acquired **(A).** The membrane is then cut in three parts, according to the molecular weight marker positions, in order to perform incubations with different antibodies (Ab) (PARP, p53 and β-actin, LC3). Following the incubation with each Ab (p53 and β-act in this figure), the chemiluminescence is acquired for protein quantification **(B)**. To get a precise report of all protein bands, after the acquisition of the chemiluminescence signal of each antibody, the U, M and B membrane portions are joined and an image of the whole membrane, merged with the molecular weight marker, is taken **(C)**.

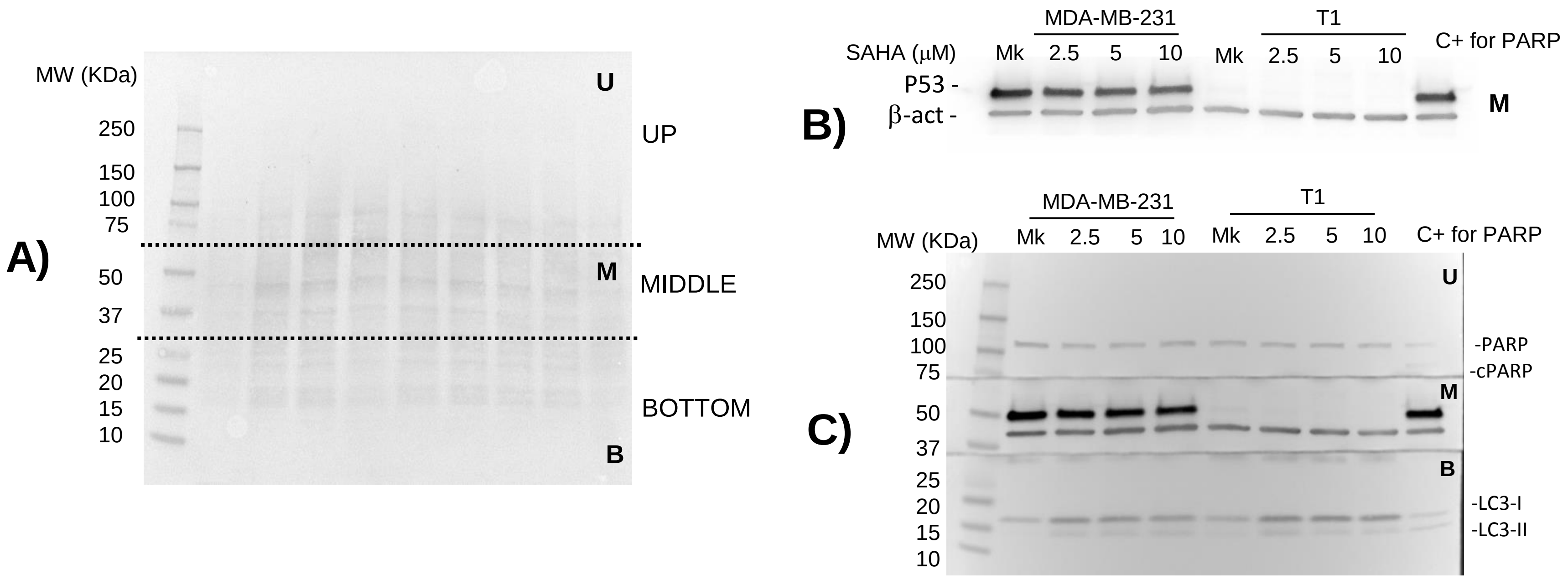

# Supplementary Figures

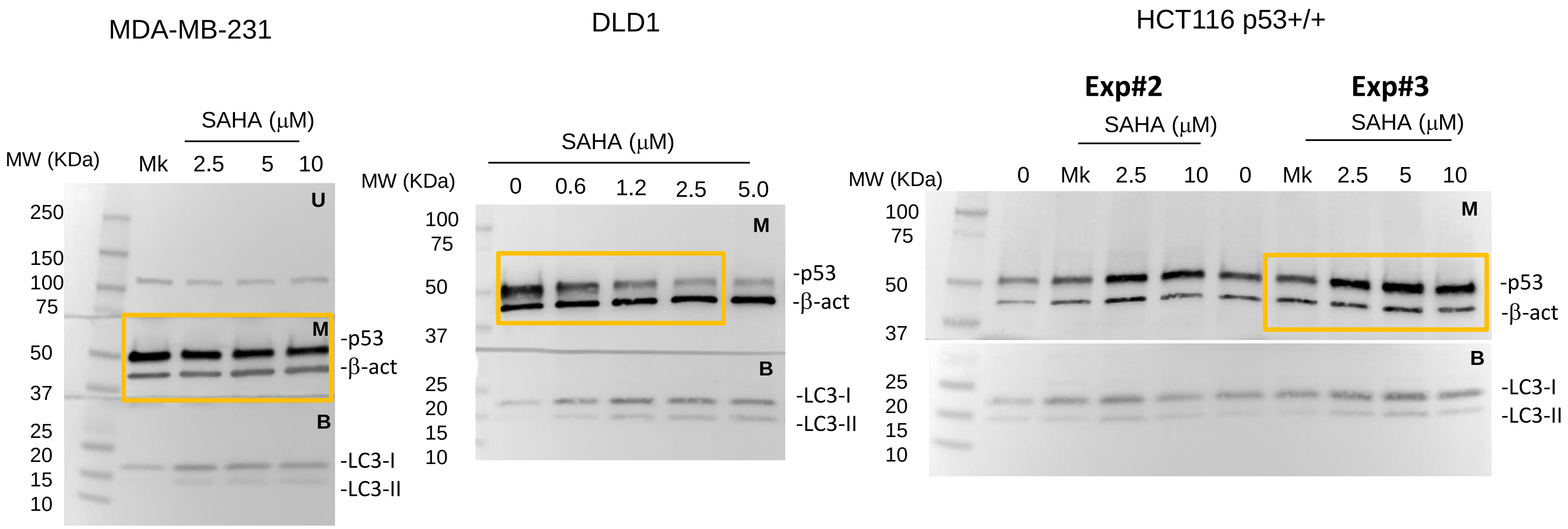


**<u>Supplementary Figure S1</u>** *<u>Full-length blots of proteins illustrated in Fig 1</u>*. For each cell lines (as indicated), the membrane loaded with protein extracts of cells treated with different SAHA concentrations were processed as described in Suppl Informations. The membranes M were first hybridized with the anti-p53 Ab (DO1) and then with the anti-β-actin Ab. After primary antibodies hybridizations, the membranes were hybridized with the common secondary Ab and the chemiluminescence from p53 and β-actin was analyzed in the same UVITEC acquisition. The membranes B were hybridized with the anti-LC3 Ab, followed by hybridization with the secondary Ab and chemiluminescence was analyzed by UVITEC. After the signal acquisition for each antibody, the M and B membranes were joined and an image of the whole membrane, merged with the molecular weight marker, was taken. The orange squares outline the cropped areas reported in Fig 1.

Fig. S1

A)

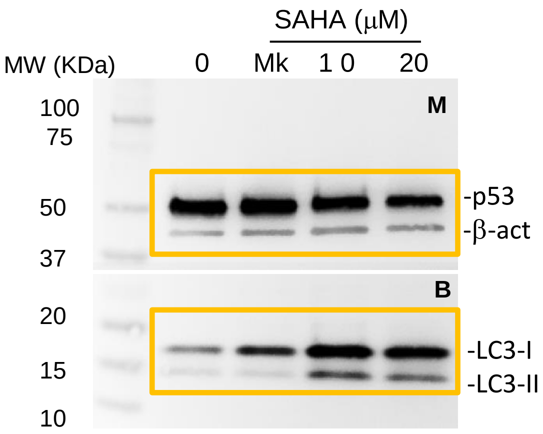


B)

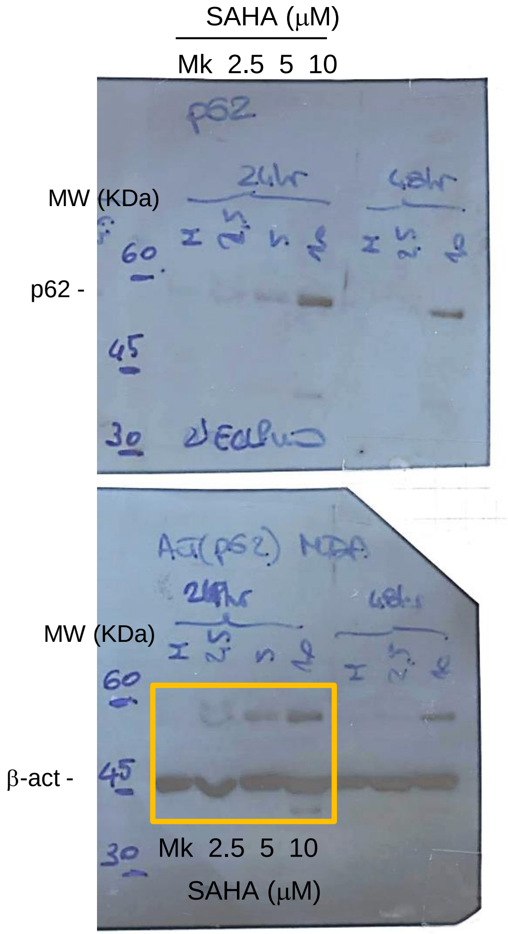


**<u>Supplementary Figure S2</u>** *<u>Full-length blots of proteins illustrated in Fig 2B</u>*. **A)** The membrane loaded with protein extracts of MDA-MB-231 cells treated with different SAHA concentrations were processed as described in Suppl Informations. The membranes M was first hybridized with the anti-p53 Ab (DO1) and then with the anti-β-actin Ab. After primary antibodies hybridizations, the membrane was hybridized with the common secondary Ab and the chemiluminescence from p53 and β-actin was analyzed in the same UVITEC acquisition. The membranes B was hybridized with the anti-LC3 Ab, followed by hybridization with the secondary Ab and chemiluminescence was analyzed by UVITEC. After the signal acquisition for each antibody, the M and B membranes were joined and an image of the whole membrane, merged with the molecular weight marker, was taken. The orange squares outline the cropped areas reported in Fig 2B, right panel; **B)** The membrane loaded with protein extracts of MDA-MB-231 cells treated with different SAHA concentrations were first hybridized with the anti-p62 Ab (Santa Cruz Biotech., sc-25575) and then with the anti-β-actin Ab. Chemiluminescence of protein bands was detected by autoradiographic film exposure. The orange square outlines the cropped area reported in Fig 2B, left panel.

Fig. S2

A) B)

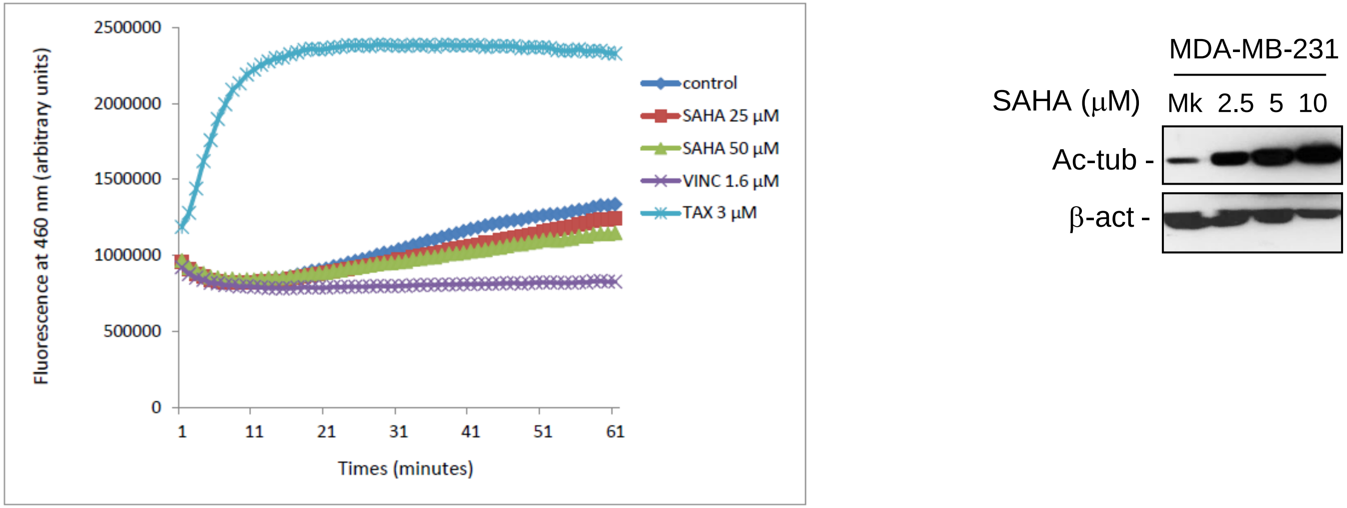


**<u>Supplementary Figure S3</u> A)** ***In vitro tubulin polymerization assay****.* The effect of SAHA on the *in vitro* assembly of tubulin microtubules was evaluated using a fluorescence-based microtubule polymerization assay kit (Cytoskeleton Inc., Denver, USA), according to the manufacturer’s protocol. Briefly, 2mg/ml porcine brain tubulin (>99% pure with minimal contamination of microtubule-associated proteins) was incubated with tubulin buffer, cushion buffer, GTP without or with 2.5-5 μM SAHA in a pre-warmed half area 96-well plate (Corning Costar) and the reaction was initiated by the addition of tubulin. Three μM Taxol and 1.5 μM Vincristine were used for enhancing or suppressing tubulin polymerisation, respectively (positive controls). The plate was incubated at 37 °C in a fluorescence microplate reader (Mithras LB 940, Berthold Technologies), and microtubule assembly was monitored by measuring the increase in fluorescence due to the incorporation of a fluorescence reporter into microtubules as polymerization proceeds. Fluorescence reading ($\lambda_{ex}$, 355 nm; $\lambda_{em}$, 460nm) was done every 5 minutes for 60 min, with 5 sec shaking every cycle. **B)** ***Increase of acetylated tubulin*** in cell extracts from SAHA-treated MDA-MB-231 cells as shown by western blot analysis.

Fig. S3

# DLD1

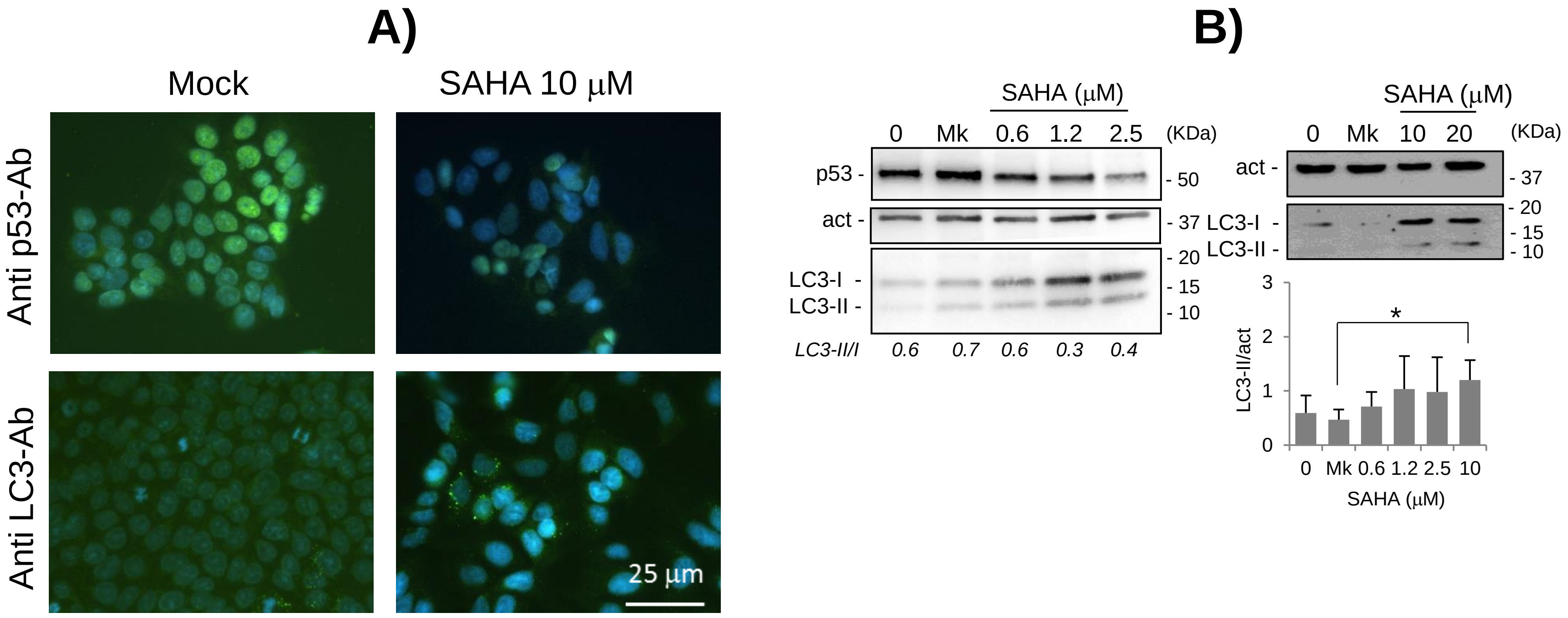


**<u>Supplementary Figure S4</u>** ***Mutp53 degradation and LC3-II increase induced by SAHA in DLD1 cells*. A)** DLD1 (p53-S241F) were mock- or SAHA-treated for 24 h and fixed for immunostaining with antibodies against p53 or endogenous LC3. Immunofluorescence and DAPI images were taken with a 40x magnification and merged; **B)** Representative western blots showing the mutp53 down-modulation and the LC3-II increase in cell extracts from SAHA-treated cells. The histogram below shows the level of LC3-II normalized for β-actin obtained after chemiluminescence analysis by UVITEC of western blots from more than three independent experiments *(*p<0.05).*

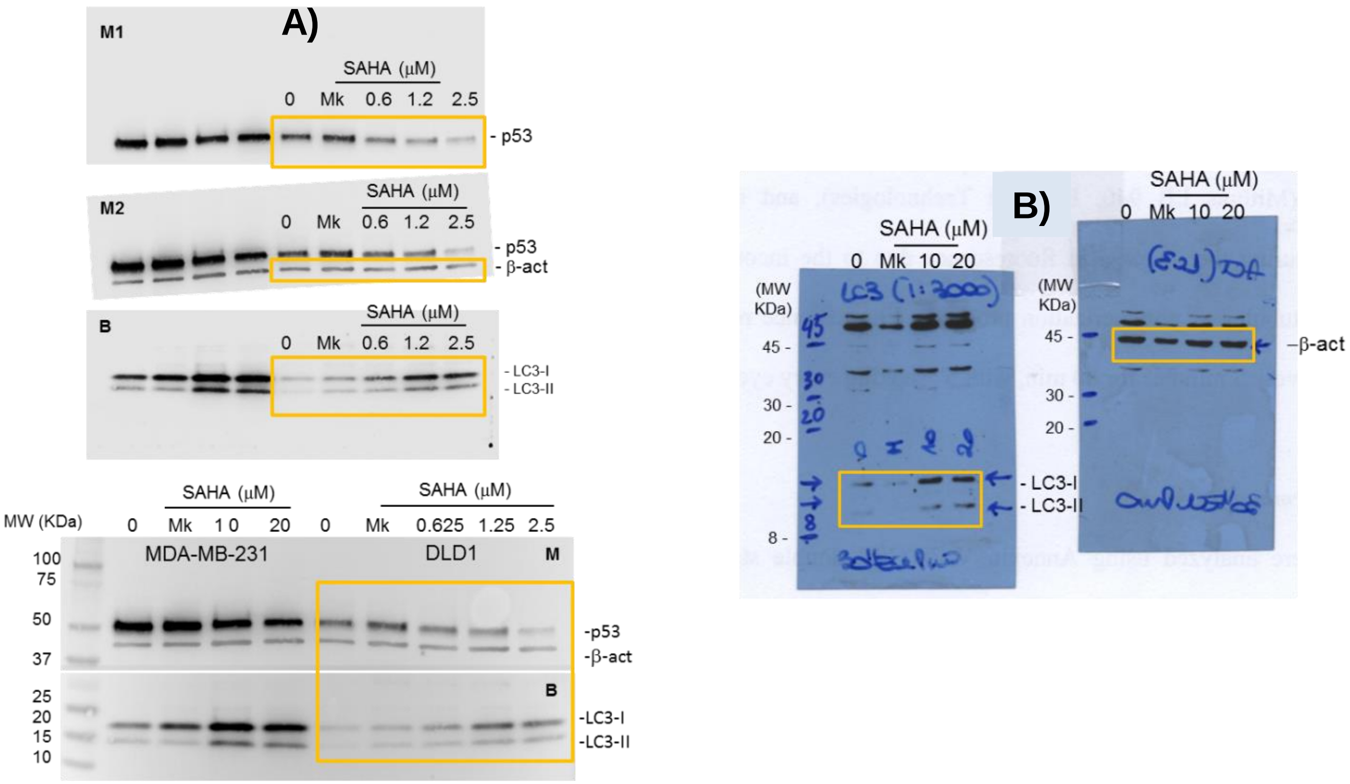


**Supplementary Figure S5** *Full-length blots of proteins illustrated in Suppl Fig S4.* The membrane were processed as described in Suppl Informations. **A)** The membrane M was hybridized with the anti-p53 Ab (DO1), the secondary Ab and the chemiluminescence from p53 was acquired by UVITEC (M1). The same membrane was then hybridized with the anti-β-actin Ab and after incubation with the secondary Ab, the chemiluminescence was again acquired with UVITEC (M2). The membranes B was hybridized with the anti-LC3 Ab, followed by hybridization with the secondary Ab and chemiluminescence was acquired by UVITEC (B). For each Ab, the first chemiluminescence acquisition is used for protein quantification and is shown on the manuscript figure. The orange squares outline the cropped areas reported in Suppl Fig S4. The image of the whole membrane (containing cell extracts from another experiment), merged with the molecular weight marker, was taken; **B)** The membrane were first hybridized with the anti-LC3 Ab (Santa Cruz Biotech., sc-25575) and then with the β-actin Ab. After hybridization with the respective secondary Abs, chemiluminescence of protein bands was detected by autoradiographic film exposure. The orange squares outline the cropped areas reported in Suppl. Fig S4.

Fig. S5

# MDA-MB-231

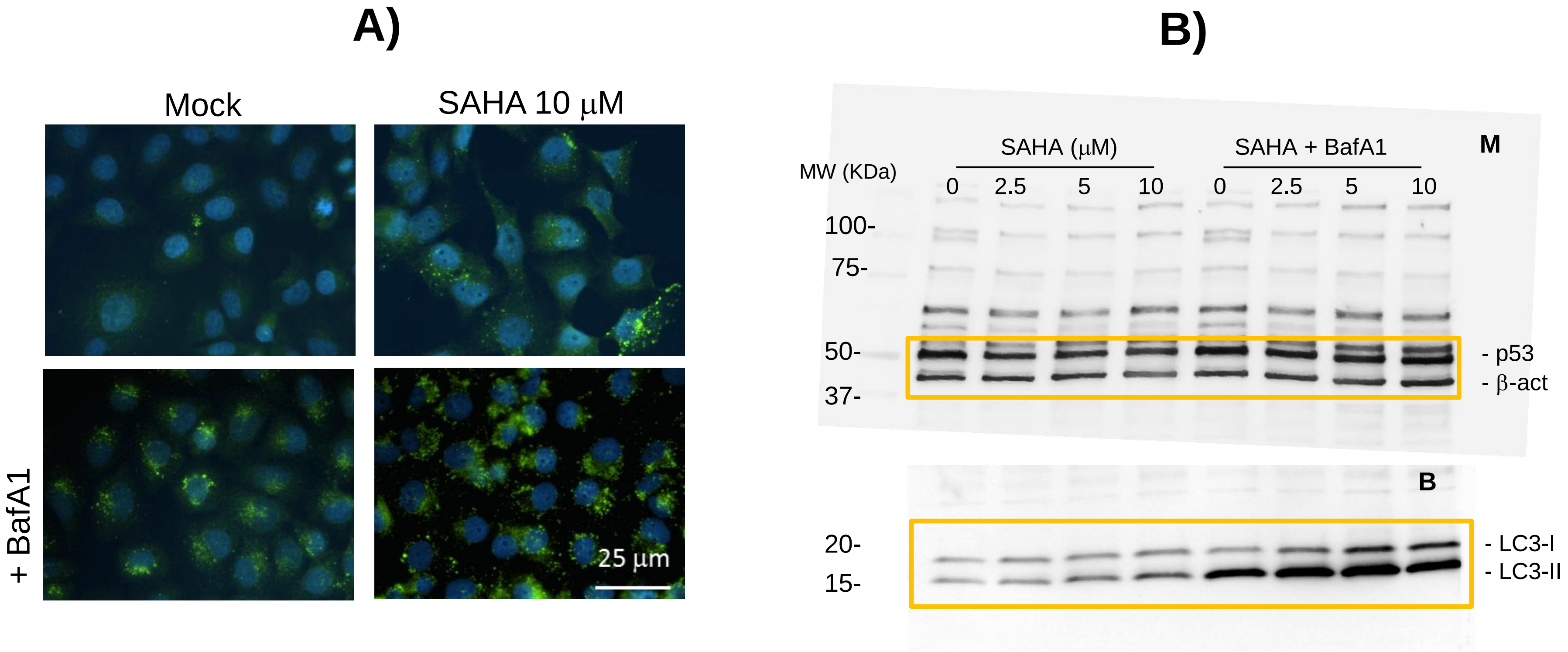


**<u>Supplementary Figure S6</u>** ***Autophagy inhibition stabilizes mutp53 in MDA-MB-231 cells*** **A)** MDA-MB-231 (p53-R280K) cells were mock or SAHA-treated for 24 h and fixed for immunostaining with antibodies against endogenous LC3. BafA1 was added in mock- and SAHA-treated cells 3 h before the withdrawal. Immunofluorescence and DAPI images were taken with a 40x magnification and merged. The addition of BafA1 3 h before the withdrawal of SAHA-treated cells caused a further increase of LC3-positive green vacuoles (compare right upper and right lower panels); **B)** *<u>Full-length blots of proteins illustrated in Fig 3A</u>*. The membrane loaded with protein extracts of MDA-MB-231 cells treated with different SAHA concentrations, with or without BafA1, were processed as described in Suppl Informations. The membranes M was first hybridized with the anti-p53 Ab (DO1) and then with the anti-β-actin Ab. After primary antibodies hybridizations, the membranes was hybridized with the common secondary Ab and the chemiluminescence from p53 and β-actin was analyzed in the same UVITEC acquisition. The membrane B was hybridized with the anti-LC3 Ab, followed by hybridization with the secondary Ab and chemiluminescence was analyzed by UVITEC. The orange squares outline the cropped areas reported in Fig 3A.

Fig. S6

**DLD1**

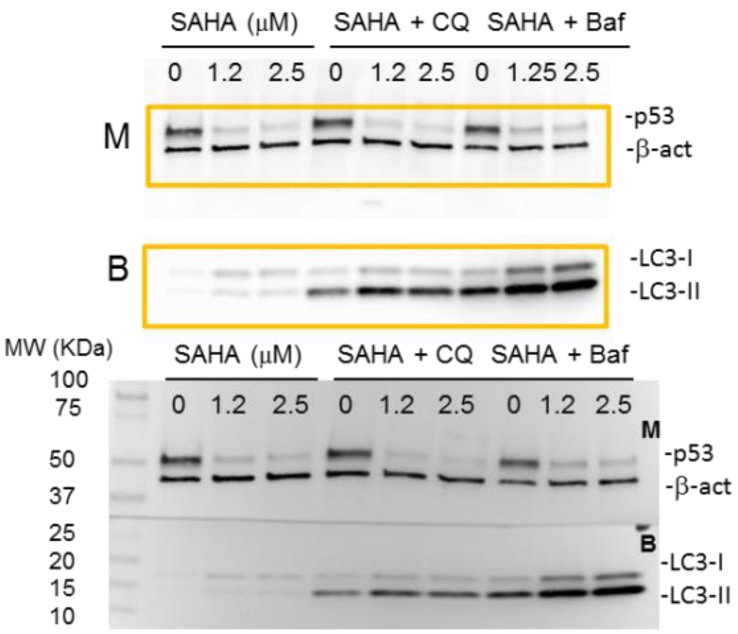


**<u>Supplementary Figure S7</u>** *<u>Full-length blots of proteins illustrated in Fig. 3C</u>*. The membrane were processed as described in Suppl Informations. The membrane M was hybridized with the anti-p53 Ab (DO1), the secondary Ab and the chemiluminescence from p53 was acquired by UVITEC. The same membrane was then hybridized with the anti-β-actin Ab and after incubation with the secondary Ab, the chemiluminescence was again acquired with UVITEC. The membranes B was hybridized with the anti-LC3 Ab, followed by hybridization with the secondary Ab and chemiluminescence was acquired by UVITEC (B). For each Ab, the first chemiluminescence acquisition is used for protein quantification and is shown on the manuscript figure. The orange squares outline the cropped areas reported in Fig 3C. After the signal acquisition for each antibody, the M and B membranes were joined and an image of the whole membrane, merged with the molecular weight marker, was taken.

Fig. S7

**A) Trypan blue assay in DLD1**

Cells were treated with different SAHA (S) concentrations with or without the addition of BafA1 (100 nM) or CQ (50 µM) for 24 h. The percentage of survival was determined by trypan blue exclusion method as blue/total cells.

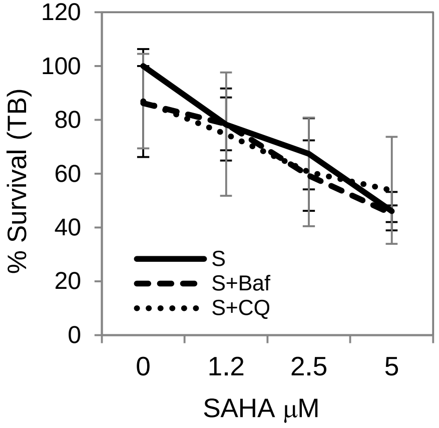


**B) xCELLigence RTCA assay in DLD1**

Cell proliferation/survival assays of SAHA-treated DLD1 cells measured by xCELLigence system; the $t_0$ arrows indicate the time of SAHA addition.

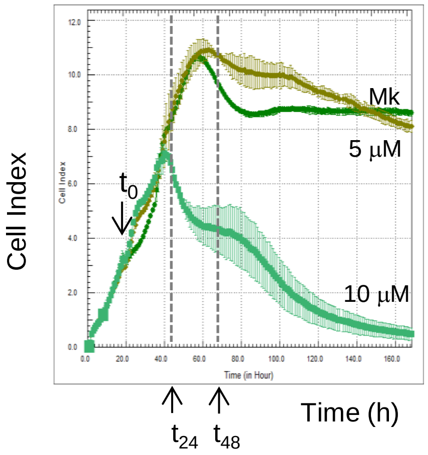


**C) Colony assay**

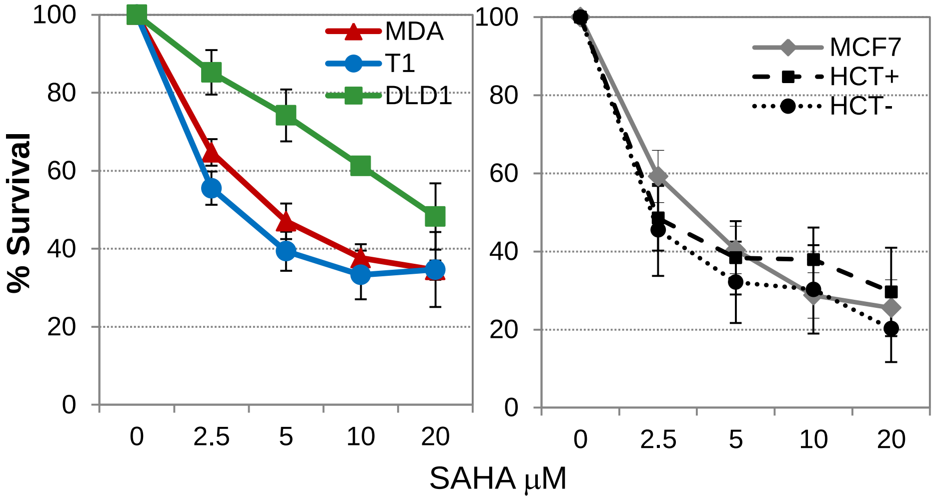


**D) MTT assay**

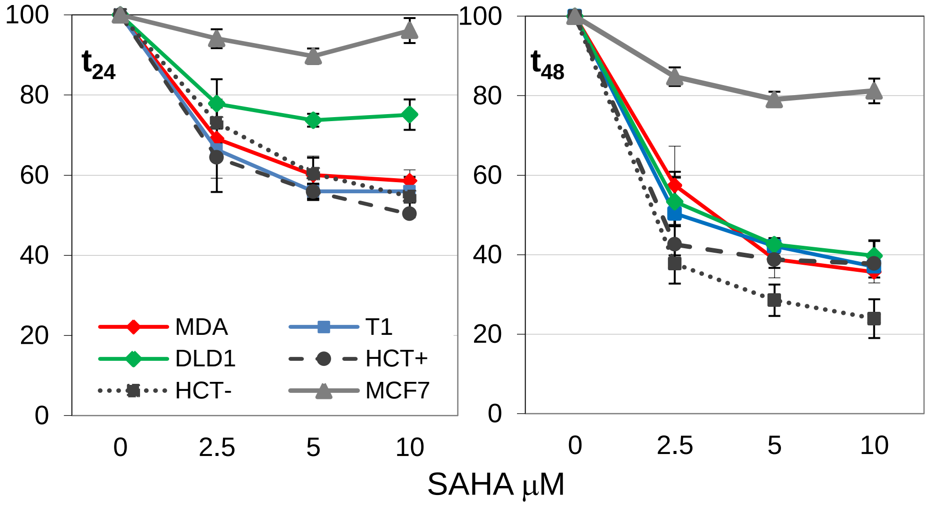


***SAHA-induced cytotoxicity measured in MDA-MB-231, T1, DLD1 MCF7, HCT116 wtp53$^{+/+}$ and HCT116 wtp53$^{-/-}$ cell lines by colony forming and MTT assays***. For colony forming assay cells were seeded at a density of 400 cells and allowed to growth for 24 h. Cells were treated by adding the drug directly in the medium at the indicated concentrations. After 24 h of treatment, the medium was removed and replaced with complete fresh medium. After 8–10 days, the colonies were counted. For MTT, cells were seeded in 96-well and treated with different SAHA concentrations in triplicates. The percentage of viable cells was determined after 24h and 48h of treatment. The average and the standard deviations of at least three independent experiments are reported.

Fig. S8

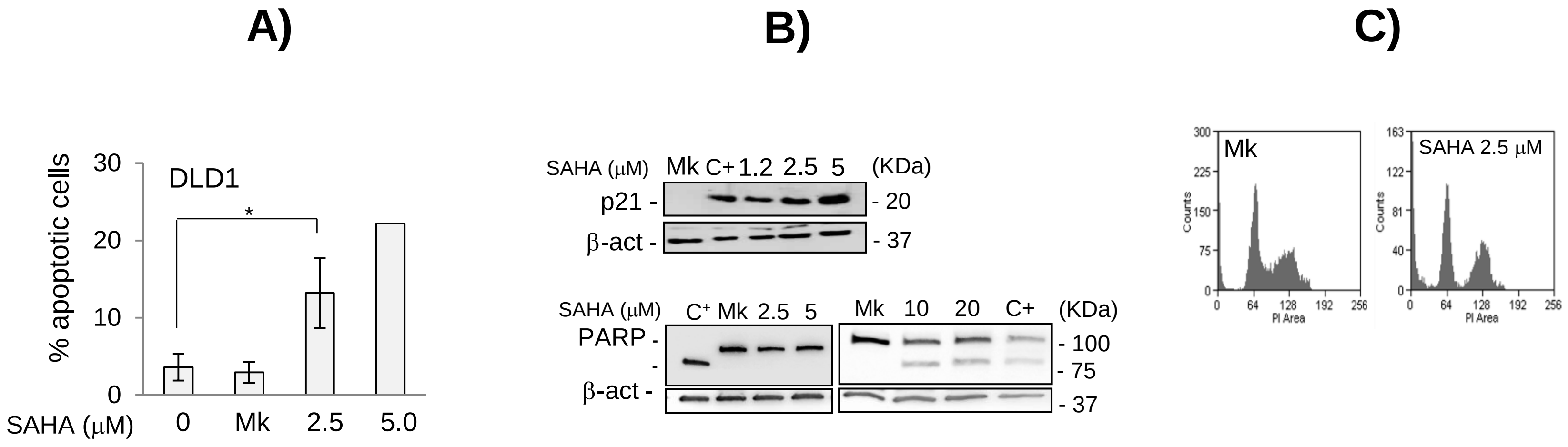


**<u>Supplementary Figure S9</u>** ***SAHA induced apoptosis and $G_2$/M arrest in DLD1 cells.*** **A**) Induction of apoptosis after SAHA treatment determined by Annexin/PI assay (*p<0.05); **B**) ) Representative western blots showing the level of p21 and PARP cleavage after SAHA treatment. (Full-length blots are presented in Supplementary Fig. S9); **C**) Representative PI cell cycle profiles of Mock and SAHA-treated DLD1 cells.

Fig. S9

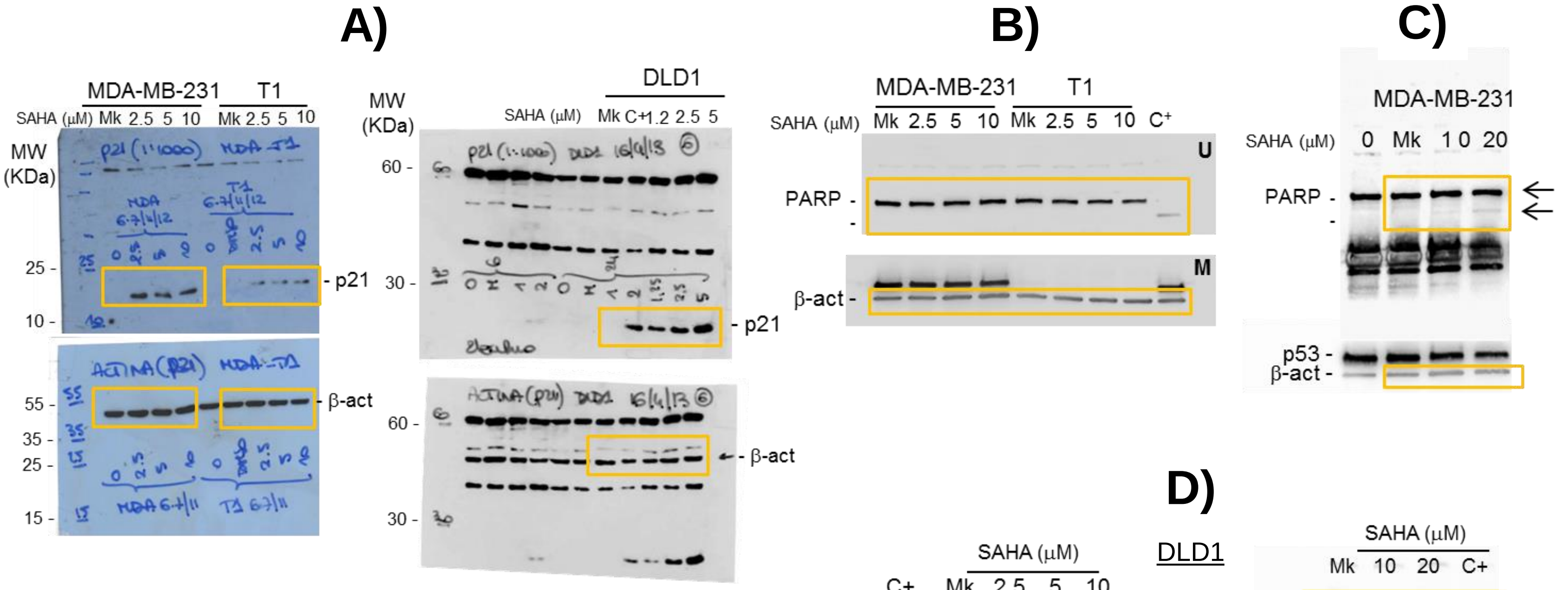


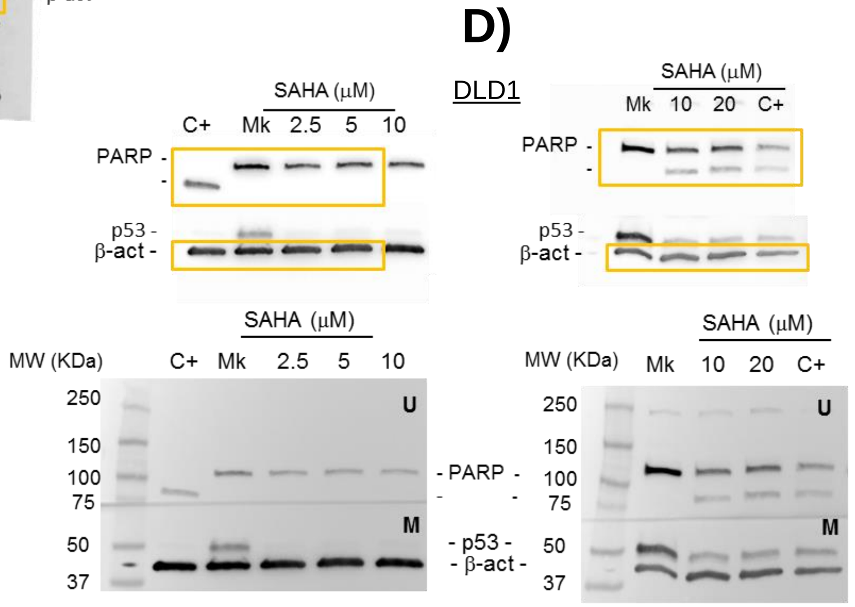


**Supplementary Figure S10** *Full-length blots of proteins illustrated in Fig 5D and Suppl Fig S9.* **A)** For each cell lines (as indicated), the membrane loaded with protein extracts of cells treated with different SAHA concentrations were first hybridized with the anti-p21 Ab and then with the anti-β-actin Ab. Chemiluminescence of protein bands was detected by autoradiographic film exposure; **B)** The membranes were processed as described in Suppl Inf: the upper membrane (U) was incubated with PARP Ab, the M membrane was incubated with p53 and β-actin Abs. Following the incubations with the respective secondary Abs, chemiluminescence was acquired. The whole membrane, merged with the molecular weight marker is reported in Suppl Informations figure; **C)** The membrane is the same as in Fig. S2A. After p53 and β-actin Abs ibridization, the membrane was incubated with PARP Ab and overexposed to see the faint band of cleaved PARP. For the whole membrane with MW marker see Fig. S2A; **D)** Membranes loaded with protein extracts (as indicated) were processed as above: the upper membranes (U) were incubated with PARP Ab, the M membranes were incubated with p53 and β-actin Abs. Following the incubations with the respective secondary Abs, chemiluminescence was acquired. The U and M membranes were joined and an image of the whole membrane, merged with the molecular weight marker, was taken. The orange squares outlines the cropped areas reported in Fig 5D.

Fig. S10

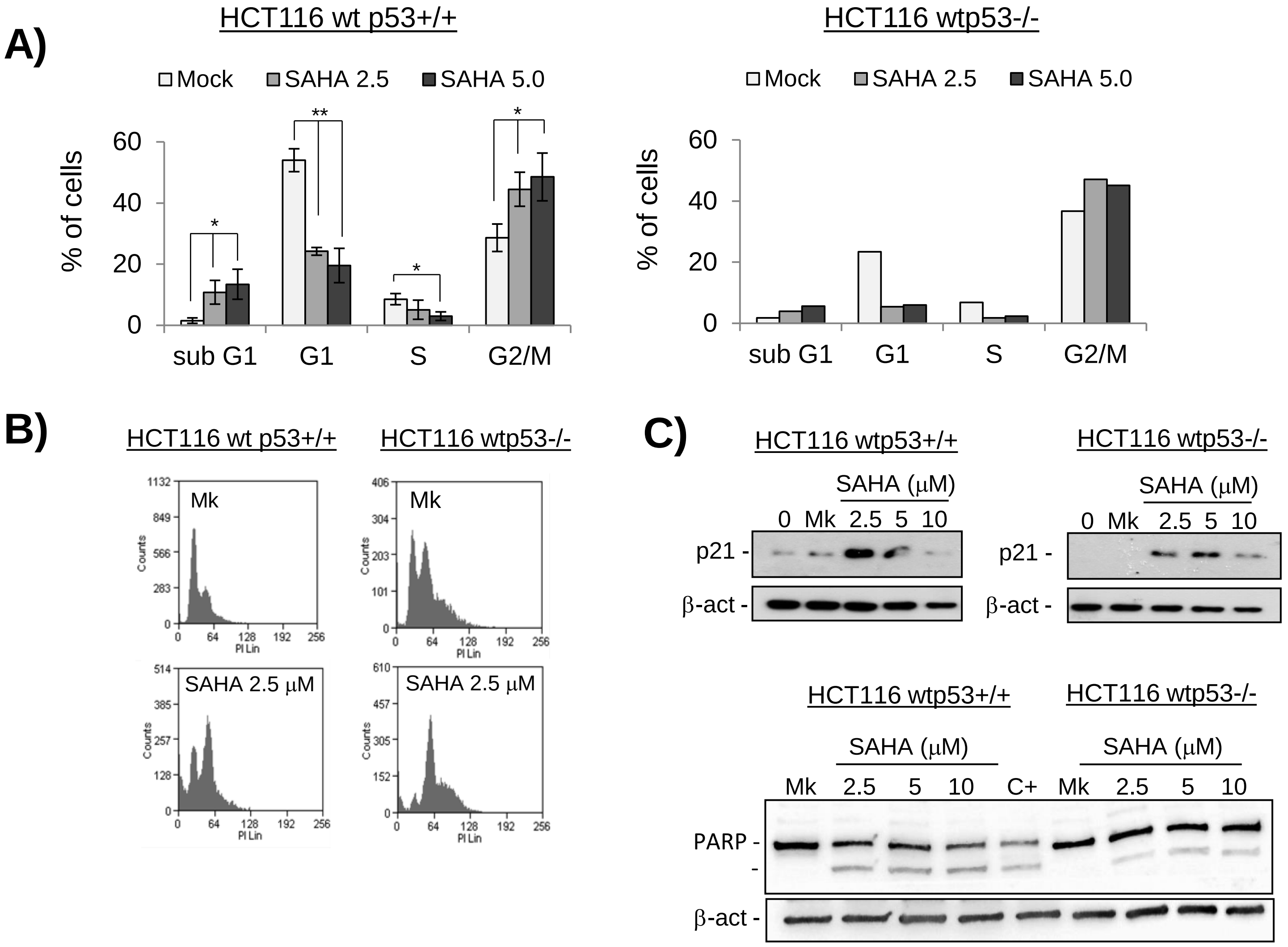


**Supplementary Figure S11** ***SAHA induced apoptosis and $G_2$/M arrest in wtp53$^{+/+}$and wtp53$^{-/-}$ HCT116 cells***. **A**) Percentage of subG1 cells and cell cycle distribution determined by PI analysis in SAHA-treated HCT116 wtp53$^{+/+}$ and HCT116 wtp53$^{-/-}$ cells. For HCT116 wtp53$^{-/-}$ cells, only one PI experiment could be analyzed; **B**) Representative PI cell cycle profiles of Mock and SAHA-treated HCT116 cells; **C**) Representative western blots showing the level of p21 and PARP cleavage after SAHA treatment. (*$p$<0.05; **$p$<0.005). Full-length blots of proteins are presented in Fig S12.

Fig. S11

**A)**

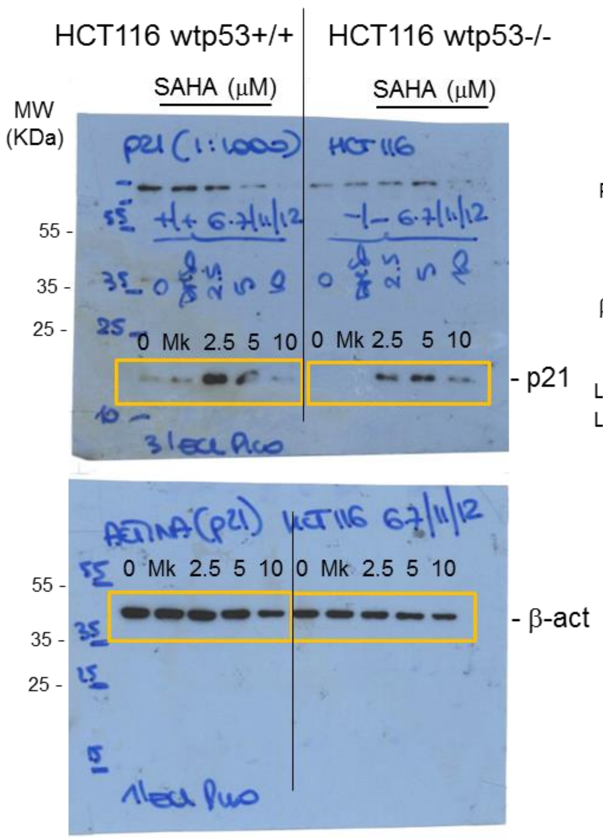


**B)**

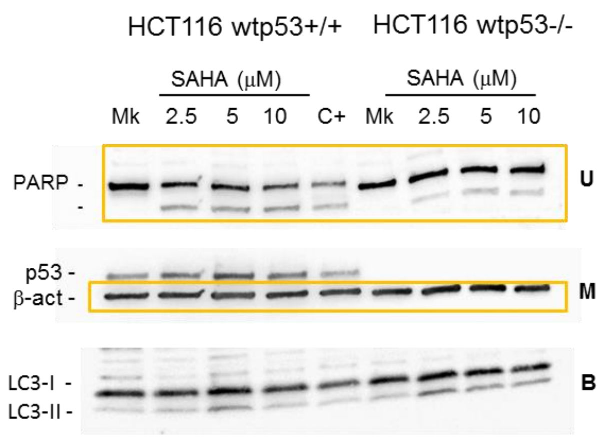


**C)**

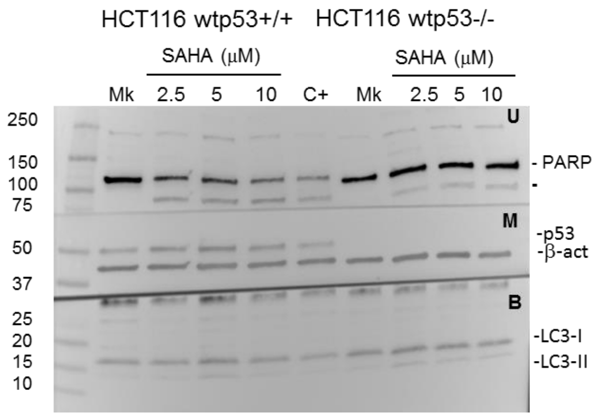


**<u>Supplementary Figure S12</u>** *<u>Full-length blots of proteins illustrated in Fig S11</u>*. **A)** Membranes loaded with cell extracts of HCT116 wtp53$^{+/+}$ and HCT116 wtp53$^{-/-}$ treated with different SAHA concentrations (as reported on the autoradiographic film) were first hybridized with p21 Ab and then with the anti-β-actin Ab. The protein molecular weights are shown on the film. The orange squares outlines the cropped areas reported in Fig S11,C;B**b)** The membrane loaded with cell extracts of HCT116 wtp53$^{+/+}$ and HCT116 wtp53$^{-/-}$ treated with different SAHA concentrations were processed as described in Suppl Informations. The membrane U was hybridized with the anti-PARP Ab, followed by hybridization with the secondary Ab; chemiluminescence was analyzed by UVITEC. The membrane M was first hybridized with the anti-p53 Ab (DO1) and then with the anti-β-actin Ab. After primary antibodies, the membrane was hybridized with the common secondary Ab and the chemiluminescence from P53 and β-actin was analyzed in the same UVITEC acquisition. The membrane B was hybridized with the anti-LC3 Ab and chemiluminescence was analyzed by UVITEC. The orange squares outlines the cropped areas reported in Fig S11,C; **C)** After the signal acquisition for each antibody, the U, M and B membranes were joined and an image of the whole membrane, merged with the molecular weight marker, was taken.

Fig. S12

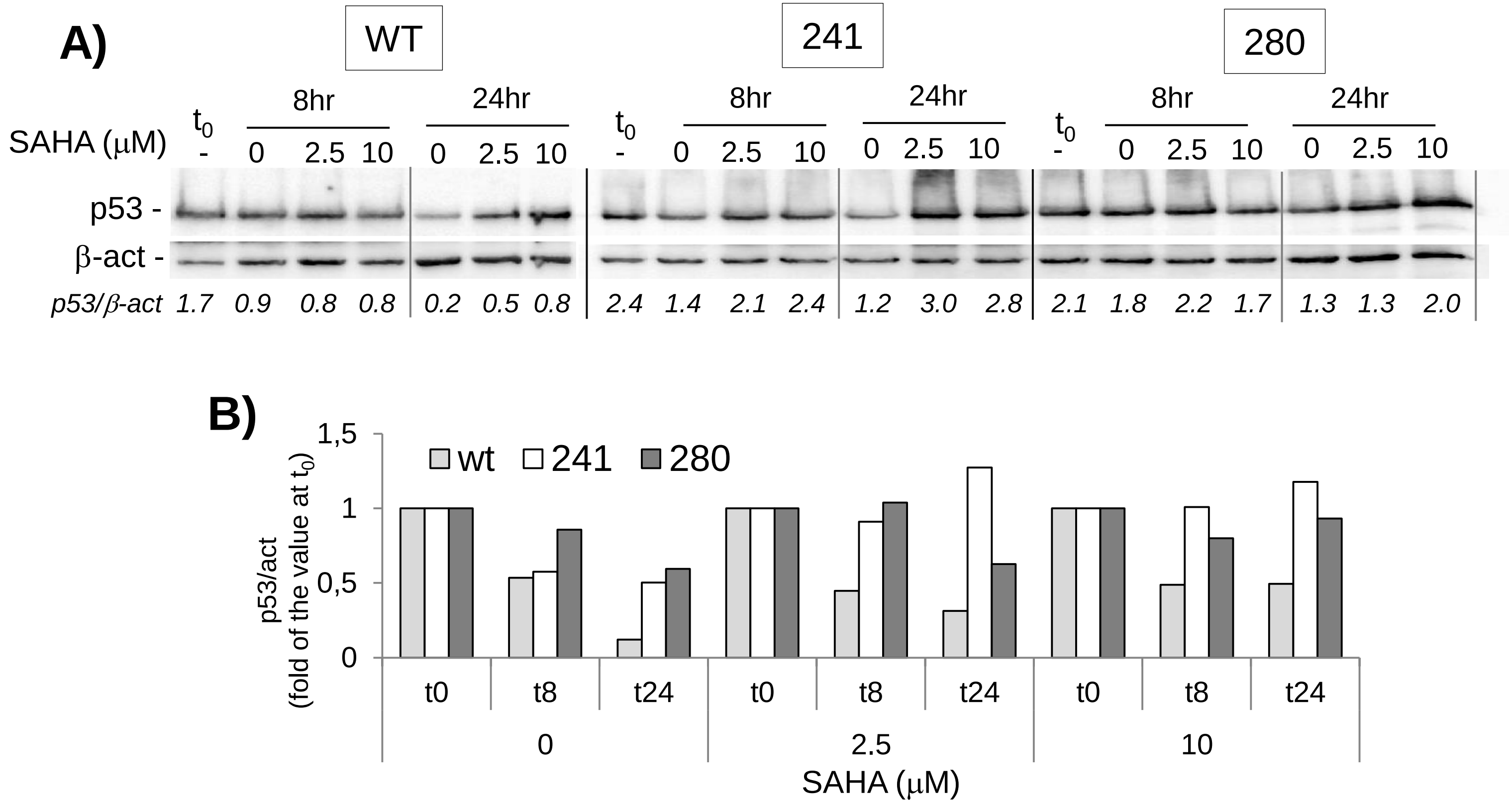


<u>**Supplementary Figure S13**</u> ***Modulation of wtp53, p53-S241F and p53-R280K proteins in transiently transfected HCT116 wtp53$^{-/-}$ cells following SAHA treatment***. **A)** HCT116 wtp53$^{-/-}$ cells were transiently transfected with plasmid expressing wtp53, p53-S241F and p53-R280K proteins as previously described (Foggetti et al., 2017). After 24 h ($T_0$), cells were treated with SAHA for 8 h and 24 h. Cells were collected and cell extracts prepared. Western blot showing the level of p53s in transfected cells treated with SAHA is reported, together with the p53/β-actin ratio calculated by UVITEC analysis. (Full-length blots of proteins are presented in Fig S14). **B)** To better highlight the modulation of wtp53, p53-S241F and p53-R280K proteins after treatment with different SAHA concentrations, a histogram was constructed by normalizing each p53/β-actin value reported above for the p53/β-actin value calculated at $T_0$.

Fig. S13

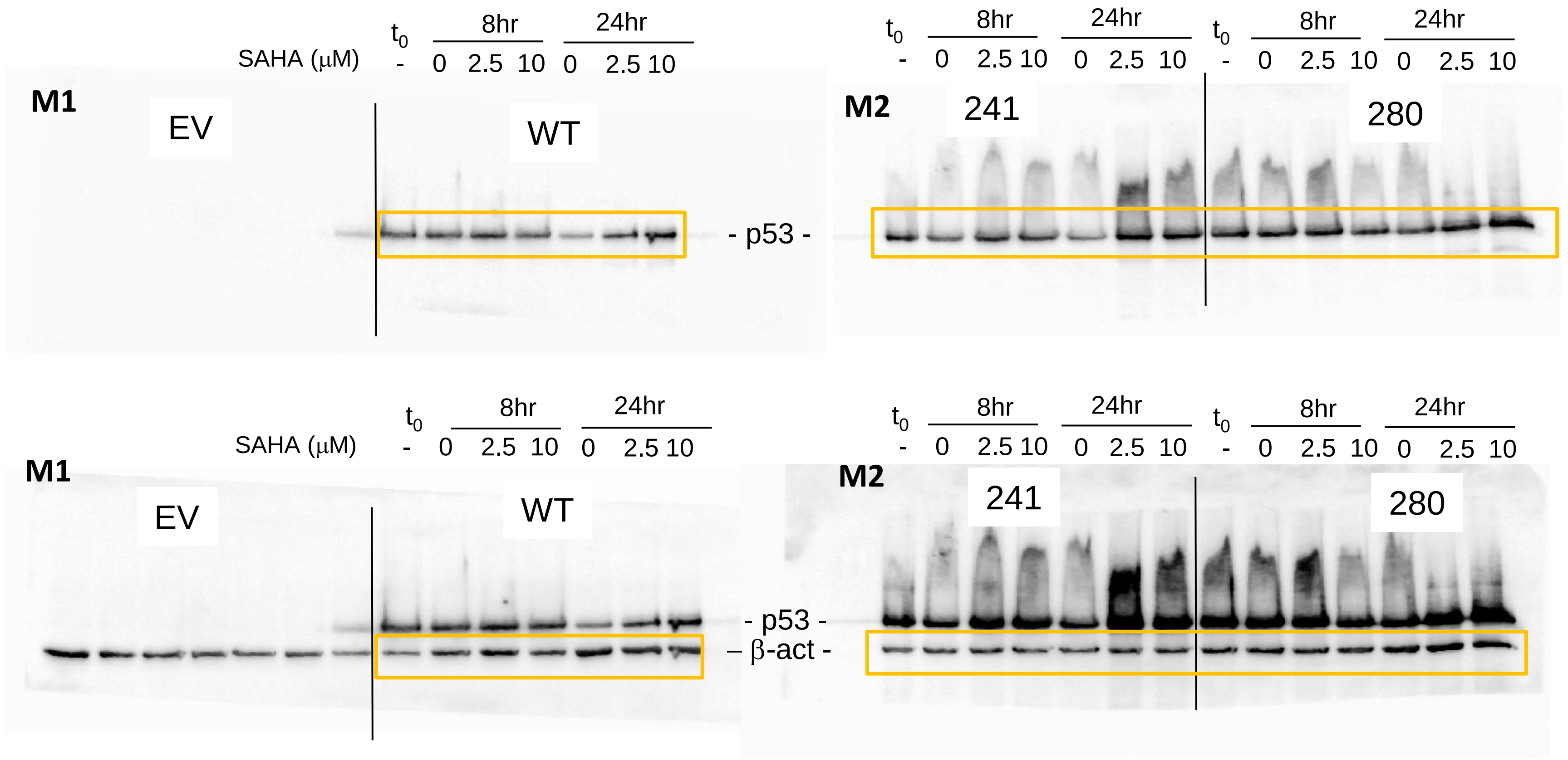


**Supplementary Figure S14** *Full-length blots of proteins illustrated in Fig S13*. Membranes (M1 and M2) loaded with cell extracts of HCT116 wtp53$^{-/-}$ cells transiently transfected with plasmid expressing wtp53, p53-S241F and p53-R280K proteins were first hybridized with the anti-p53 Ab (DO1) and the chemiluminescence was acquired with UVITEC. Then the same membranes were incubated with the anti-β-actin Ab and after incubation with the secondary Ab, the chemiluminescence for β-actin was acquired. The orange squares outlines the cropped areas reported in Fig S13.

Fig. S14

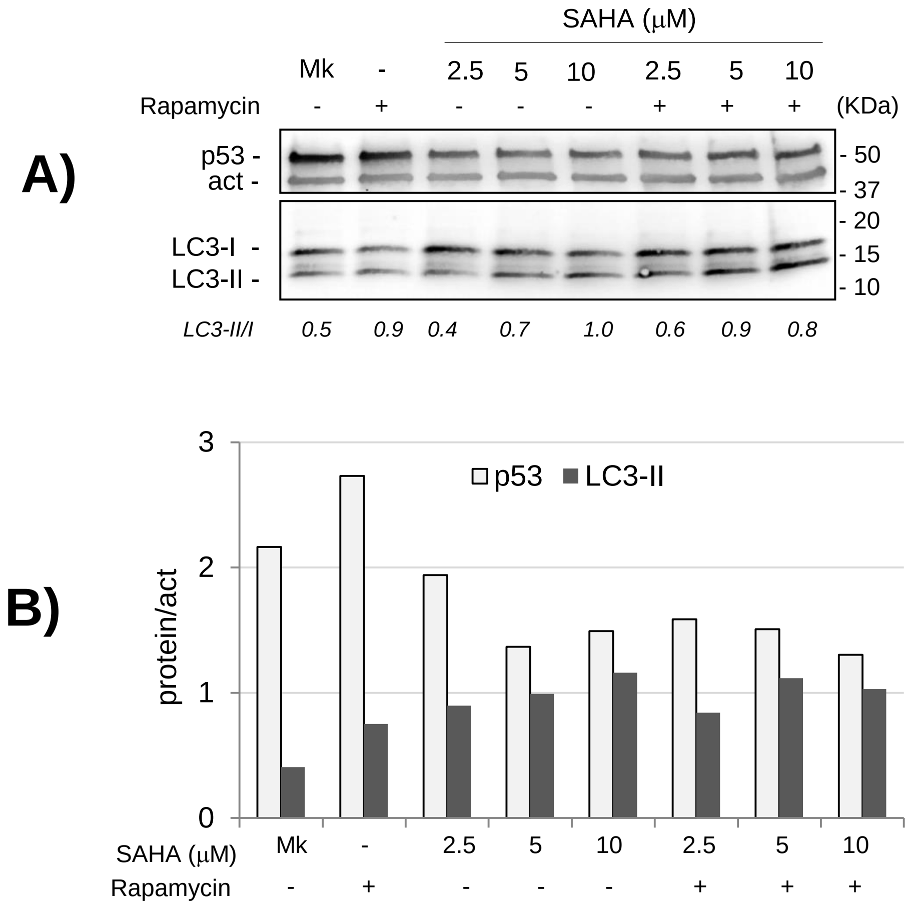


**<u>Supplementary Figure S15</u>** ***Mutp53 and LC3-II protein level in MDA-MB-231 cells treated with 100nM Rapamycin, and SAHA, alone and in combination. A)*** Representative western blots showing the mutp53 down-modulation and the LC3-II increase in cell extracts from SAHA-treated cells. The combined SAHA+ rapamycin treatment did not affect the level of mutp53 in comparison with SAHA alone; **B)** To better highlight the results, the histogram shows the level of LC3-II and p53 normalized for β-actin obtained after chemiluminescence analysis by UVITEC of western blots.

Fig. S15
